# Impacts of turmeric and its principal bioactive curcumin on human health: Pharmaceutical, medicinal, and food applications: A comprehensive review

**DOI:** 10.3389/fnut.2022.1040259

**Published:** 2023-01-10

**Authors:** Mohamed T. El-Saadony, Tao Yang, Sameh A. Korma, Mahmoud Sitohy, Taia A. Abd El-Mageed, Samy Selim, Soad K. Al Jaouni, Heba M. Salem, Yasser Mahmmod, Soliman M. Soliman, Shaimaa A. A. Mo’men, Walid F. A. Mosa, Nahed A. El-Wafai, Hamed E. Abou-Aly, Basel Sitohy, Mohamed E. Abd El-Hack, Khaled A. El-Tarabily, Ahmed M. Saad

**Affiliations:** ^1^Department of Agricultural Microbiology, Faculty of Agriculture, Zagazig University, Zagazig, Egypt; ^2^Key Laboratory of Tropical Translational Medicine of Ministry of Education, School of Pharmacy, Hainan Medical University, Haikou, China; ^3^Department of Food Science, Faculty of Agriculture, Zagazig University, Zagazig, Egypt; ^4^Department of Biochemistry, Faculty of Agriculture, Zagazig University, Zagazig, Egypt; ^5^Department of Soils and Water, Faculty of Agriculture, Fayoum University, Fayoum, Egypt; ^6^Department of Clinical Laboratory Sciences, College of Applied Medical Sciences, Jouf University, Sakaka, Saudi Arabia; ^7^Department of Hematology/Oncology, Yousef Abdulatif Jameel Scientific Chair of Prophetic Medicine Application, Faculty of Medicine, King Abdulaziz University, Jeddah, Saudi Arabia; ^8^Department of Poultry Diseases, Faculty of Veterinary Medicine, Cairo University, Giza, Egypt; ^9^Department of Veterinary Sciences, Faculty of Health Sciences, Higher Colleges of Technology, Al Ain, United Arab Emirates; ^10^Department of Medicine and Infectious Diseases, Faculty of Veterinary Medicine, Cairo University, Giza, Egypt; ^11^Department of Entomology, Faculty of Science, Ain Shams University, Cairo, Egypt; ^12^Plant Production Department (Horticulture-Pomology), Faculty of Agriculture Saba Basha, Alexandria University, Alexandria, Egypt; ^13^Department of Agricultural Microbiology, Faculty of Agriculture, Benha University, Benha, Egypt; ^14^Department of Clinical Microbiology, Infection and Immunology, Umeå University, Umeå, Sweden; ^15^Department of Radiation Sciences, Oncology, Umeå University, Umeå, Sweden; ^16^Department of Poultry Diseases, Faculty of Agriculture, Zagazig University, Zagazig, Egypt; ^17^Department of Biology, College of Science, United Arab Emirates University, Al Ain, United Arab Emirates; ^18^Khalifa Center for Genetic Engineering and Biotechnology, United Arab Emirates University, Al Ain, United Arab Emirates; ^19^Harry Butler Institute, Murdoch University, Murdoch, WA, Australia

**Keywords:** bioavailability, cancer, curcumin, dietary additives, herbal treatment, polyphenolic pigment, metabolism

## Abstract

The yellow polyphenolic pigment known as curcumin, originating from the rhizome of the turmeric plant *Curcuma longa* L., has been utilized for ages in ancient medicine, as well as in cooking and food coloring. Recently, the biological activities of turmeric and curcumin have been thoroughly investigated. The studies mainly focused on their antioxidant, antitumor, anti-inflammatory, neuroprotective, hepatoprotective, and cardioprotective impacts. This review seeks to provide an in-depth, detailed discussion of curcumin usage within the food processing industries and its effect on health support and disease prevention. Curcumin’s bioavailability, bio-efficacy, and bio-safety characteristics, as well as its side effects and quality standards, are also discussed. Finally, curcumin’s multifaceted uses, food appeal enhancement, agro-industrial techniques counteracting its instability and low bioavailability, nanotechnology and focused drug delivery systems to increase its bioavailability, and prospective clinical use tactics are all discussed.

## Introduction

Medicinal herbs could be the best source for various medicines (1). Due to their different therapeutic properties, medicinal herbs have been considered by many researchers worldwide (2). In modern medicine, several studies have been conducted to find the potential effects of various extracts of medicinal herbs that have a pivotal role in the health of people and animals (3–5). Plant-based drugs may be much more appropriate in biochemical terms when compared to synthetic drugs. However, modern medicine does not essentially support natural products for medicinal uses (6–8), such as growth inhibitors of some tumors (9). Moreover, different compounds (including colchicine, vincristine, vinblastine, podophyllotoxin, and taxol) have been isolated from various herbs, and have been used against different types of tumors (10).

One of the most studied medicinal herbs is turmeric. Turmeric is the dried rhizome powder of the *Curcuma longa* plant, composed of many phytochemicals (11, 12). Concerning the approximate composition, turmeric is composed of water (80–90%), followed by carbohydrates (around 13%), proteins (2%), minerals (2%), and lipids (<1%) (13). Among the minor components of turmeric, curcuminoids have a central role and may compose up to 10% of dry turmeric powder. This category mainly comprises curcumin, dimethoxy-curcumin, and bisdemethoxycurcumin, which can compose 62–90, 9–23, and 0.3–14 mg/g of commercial turmeric products (extracts and powders), respectively. Additionally, more than 50 curcuminoids (such as bisabocurcumin, curcumalongin, cyclocurcumin, and terpecurcumin) have been identified in turmeric (14, 15), which produce the yellow color of turmeric.

While turmeric, one of the basic elements of curry powder, is used as a spice in the west, it has been used as a natural remedy in Asia for a long period, e.g., for the treatment of stomach and liver problems. Alternatively, turmeric, which entered Turkish cuisine in the 16th century, was used as a natural dye to give yellow color to the saffron rice dessert. Because of the dye substance’s resemblance to saffron, it is used in the production of zerde as a low-price alternative. Even though the first application of turmeric in history was as a dyestuff for fabrics and yarns, its current use for health purposes has largely surpassed any other application, including spices (16).

New uses of turmeric shifted from textile dying and ancient proposed medicinal properties to exploring potential health effects, including anti-carcinogenic, anti-inflammatory, anticoagulant, antimicrobial, and antioxidant impacts (17). The medicinal properties of turmeric over the centuries have had many proposed benefits, such as aiding in wound healing, allergy, asthma, sinusitis, hepatic, and heart diseases (18). Few studies have examined the whole turmeric root as an agent to help control inflammation or other health concerns. Turmeric is a healthy and safe space for reasonable consumption (19).

The most significant curcuminoid, to which the most healing properties of turmeric are attributed, is curcumin, which was first extracted from turmeric in 1815, and its molecular formula was discovered in 1910. Curcumin, with the molecular formula C_21_H_20_O_6_ and the chemical name diferuloylmethane, is the most considerable molecule isolated from plants that have been investigated in recent years (12). This molecule is inherently hydrophobic and does not dissolve in water but in substances such as dimethyl sulfoxide, acetone, ethanol, and oil (20). Turmeric contains about 3–8% curcumin (depending on the growing season). A dessert spoon of turmeric powder (an average of 3 g) will contain an average of 30–90 mg of curcumin, although other plant species also contain some curcumin (21). In various research and studies, turmeric’s antiseptic, anti-inflammatory and antioxidant properties have been proven and proposed as a complementary treatment for Alzheimer’s, diabetes, asthma, stomach ulcers, etc. (22). Over the few current decades, considerable studies have been conducted on curcumin due to its beneficial health properties, including potent antioxidant properties (23, 24), antimicrobial (25), anti-inflammation (26), anticancer effects (27), cardio-protectiveness (28), and hypoglycemic action (29).

In this review, to collect the data, we used keywords such as “turmeric,” “curcumin,” “antioxidant,” “anticancer,” “antimicrobial,” “bioavailability,” and “Food applications,” on the web of science, ekb.eg, and Google Scholar, which has a total of 294 articles between 2004 and 2023.

Despite numerous cells, culture studies indicating that low doses of curcumin were sufficient to exhibit its biological action, many animal and clinical studies revealed that a hefty dose of curcumin is necessary to trigger its full impact since its poor bioavailability restricts its bio-efficacy. Therefore, this review concentrates on the way to increase curcumin bioavailability and its impacts of turmeric and curcumin on human health, metabolism, mechanisms of action, and their usage limits and food applications.

## Extraction of bioactive compounds from turmeric

Obtaining curcuminoid-rich extracts is a crucial step in maximizing the utilization of turmeric in meals. Numerous studies have assessed the impact of traditional and innovative extraction methods (Table 1). Curcumin is the most important of turmeric’s medicinal components and makes up between 1 and 7 percent of the root (30). In terms of traditional solvent extraction, the recovery of curcumin employing a 70% hydroethanolic solution reached 69.1% (31). Due to its limited water solubility, curcumin must be extracted using organic solvents. In this context, solvent composition plays a crucial role in maximizing the extraction yield, and ethanol and acetone are the solvents of choice. In terms of the technique to separate curcumin from turmeric, continuous Soxhlet extraction might be recommended as a first step to increase the recovery of curcumin (1.3 and 6.9 g/100 g) from turmeric (32).

**TABLE 1:** List of extraction methods for bioactive compounds in turmeric.

Extraction method	Conditions	Curcumin yield	References
Conventional solvent extraction	Solid/solvent ratio: 1:6 g/g for 12 and 24 h at 50°C	69.1%	(31)
Soxhlet extraction	Sample amount: 15 g in acetone at 60°C for 8 h	6.9 g curcumin/100 g sample	(30, 32)
	10 g sample in 250 mL of ethanol for 8 h	1.3 g curcumin/100 g sample	
Ultrasound- assisted extraction	Solid/solvent ratio: 1:20 g/mL; solvent: acetone for 40 min at 40°C	3.9 g curcumin/100 g sample	(30, 32)
	Solid/solvent ratio: 1:3 g/L in acetone for 5 min	71.4%	
	Solid/solvent ratio 1:15–1:55; time: up to 1 h; solvent: ethanol, methanol, acetone, and ethyl acetate at 25–55°C	72%	
Microwave- assisted extraction	Solid/solvent ratio: 1:20 g/mL; in acetone for 0.5–3 min	2.4 g curcumin/100 g sample	(30, 34)
	Solid/solvent ratio: 1:3 g/L; in ethanol, acetone, water for 1–7	90%	
Subcritical water extraction	Sample amount: 0.1 g; flow rate: 0.5 mL/min; 5.0 MPa; temperature: 180, 200, and 220°C; and pH: 1, 1.5, and 2	1.35 g curcumin/100 g	(33, 35)
	Flow rate: 1, 2.5, and 4 mL/min; temperature: 90, 120, and 150°C; and particle size: 0.5, 1, and 1.5 mm	90%	
	Particle size: 0.6,0.71, 1, and 2 mm; temperature: 120–160°C; time: 6, 10, 14, 18, and 22 min	76%	
Supercritical carbon dioxide	10 g sample in ethanol, time: 240 min; pressure: 30 MPa; temperature: 50°C; flow rate 5 mL/min		(34)

Research utilizing innovative and environmentally friendly methods, such as water extraction, demonstrated high curcumin recovery yields (between 76.0 and 90.6%) to increase curcumin extraction’s efficacy (33). Concerning the influence of supercritical carbon dioxide (SC-CO_2_) on curcumin extraction, Wakte et al. (34) attained a recovery efficiency of 69.4% by utilizing ethanol as a co-solvent and maintaining an extraction temperature of 50°C.

Considering these technologies and methods of extracting curcumin from turmeric, both traditional and developing technologies can be applied to create curcumin-rich extracts. Considering the lengthy extraction periods to obtain the extracts using conventional technologies (agitation and Soxhlet) and the use of organic solvents (particularly acetone), the use of alternative technologies and approaches that produce extracts using a less harmful and toxic solvent in a shorter period can be of great value to increase the use of curcumin in scientific studies and commercial applications in the food and pharmaceutical industries (33, 35).

## Increasing curcumin bioavailability

Low bioavailability of any pharmaceutical agent within the body is due to; (1) poor gastrointestinal absorption, (2) high rates of metabolism, (3) inactivity of metabolic products, and (4) rapid elimination and clearance (36). Because of its tautomeric structure, high-molecular-weight, and aromatic groups, curcumin is extremely hydrophobic and, therefore, only partially absorbed through the gastrointestinal epithelium (37). One of the first studies to report this constraint, identified virtually undetectable plasma curcumin levels following a 1 g/kg oral dose in rats. Once absorbed, the liver predominantly metabolizes curcumin to form glucuronide and sulfate conjugates, representing about 99% of plasma curcumin (38). These metabolites have been reported to have low bioactivity compared to free curcumin (39). Lastly, the brief half-life of curcumin plays a crucial role in its low bioavailability. In rats, orally delivered curcuminoids reach a peak plasma concentration at 0.83 ± 0.05 h, with an elimination half-life of 1.70 ± 0.58 h (40).

Several delivery techniques have been developed to overcome the pharmaco-kinetics predisposing to poor bioavailability of orally ingested curcumin, including adjuvants, nanoparticles, liposomes, and a self-nanoemulsifying drug delivery system (SNEDDS) (41). The *in vivo* response has been varied (41), with several products posing a risk for drug–drug interactions due to their inhibition of the *P*-glycoprotein and CYP3A4 systems (42).

Regardless, curcumin’s substantial limitations have not stopped scientists from researching and enhancing its potential. Rather, they have created greater scope for developing innovative solutions to address issues with the native form. Curcumin formulations have come about because of research to improve bioavailability, permeability, circulation, half-life, and withstand metabolic processes. These formulations admit chemical curcumin derivatives and analogs with metabolic adjuvants, nanoparticles, liposomes, micelles, nanostructured lipid carriers (NLC), and phospholipid complexes (43).

Phospholipid complexes appear to have been effectively released into the worldwide market among the formulations that emerged from Longvida (Verdure Sciences, Noblesville, IN, USA), and Meriva (Indena, Milan, Italy). Longvida^®^ with the bioavailability of a solid lipid formulation containing roughly 80 mg of curcumin was observed to be four times better than that of curcumin alone. Daily therapy with 400 mg of Longvida^®^ for 4 weeks in an aged population resulted in better cognitive capabilities (44). Meriva^®^, another proprietary curcumin formulation from Indena, is used to treat osteoarthritis, diabetes mellitus, and microangiopathy in a 1:2 weight ratio of curcumin and soy lecithin (44).

**FIGURE 1:**
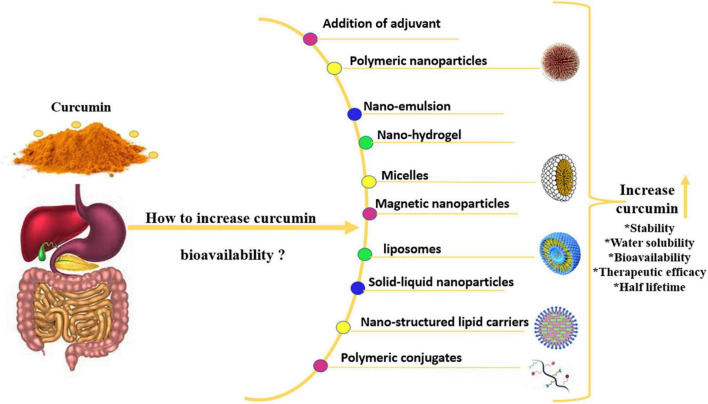
Novel methods to increase curcumin bioavailability.

Compared to Meriva, normal curcuminoid combination absorption was 29-fold lower in clinical studies. However, the C95 formulation contained 95% curcuminoid powder, which increased bioavailability fivefold. The curcumin cyclodextrin complex (CCC) is said to have a 45-fold higher bioavailability than C95 (45). The curcuminoid phospholipid complex (CPC) demonstrated a 20-fold better than curcumin bioavailability and 30-fold higher than total curcuminoids (46). Furthermore, Zeng et al. (47) examined the effect of piperine pre-administration on oral curcumin bioavailability. In this investigation, rats were given 20 mg/kg piperine first, followed by 200 mg/kg curcumin at intervals of 0.5–8 h after piperine treatment. The pre-treatment with piperine before curcumin administration significantly increased curcumin oral bioavailability in all tested rats. Curcumin permeability rose 1.85-fold when quercetin and resveratrol were combined, suggesting that resveratrol and quercetin had a cumulative impact on curcumin absorption (48). Other recent trends to improve oral bioavailability of curcumin are presented in Figure 1. Below is some recent techniques to increase the curcumin bioavailability.

### LipiSperse

LipiSperse^®^ is a novel delivery system designed to improve crystalline lipophilic agent dispersion in aqueous environments. Lipophilic active ingredients provide challenges from a formulation and bioavailability perspective. Often, improving bioavailability leads to a decreased dynamic load in final formulations (49).

LipiSperse^®^ is a mixture of surfactants, polar lipids, and solvents specifically chosen for their ability to embed into the lipophilic crystal structure of the active ingredient while keeping the hydrophilic head on the surface, increasing the hydration of the crystal by lowering the surface tension, which allows it to disperse in water. Once dispersed in water, LipiSperse^®^ then goes on to prevent the crystals from agglomeration (49).

### Employing adjuvants

Although curcumin has several benefits, many preclinical studies have demonstrated that it cannot be utilized to treat human disorders due to its low bioavailability. Utilizing adjuvants to block detoxifying enzymes shown in curcumin metabolism is one of the most important methods for enhancing the oral bioavailability of curcumin (50). Among adjuvants, black pepper piperine is one of the most effective boosters of curcumin bioavailability. The simultaneous administration of curcumin and piperine to humans or animals boosted the serum levels by more than a thousandfold (51). Additionally, epigallocatechin-3-gallate (EGCG) was utilized as an adjuvant to curcumin, increasing the bioavailability of curcumin by many orders of magnitude (52). However, the adjuvants are not restricted to the substances listed above.

Several adjuvants have been employed to enhance curcumin’s oral bioavailability for various therapeutic uses, including cancer. Recent research suggests that oral administration of curcumin and piperine for symptomatic COVID-19 therapy might dramatically reduce mortality and morbidity (53). The conjugation between piperine and curcumin may be a safe and natural option for preventing post-COVID symptoms. According to another study, with curcumin nanoparticles, prolonged topical administration and improved bioavailability of curcumin were obtained, including the possibility of skin discoloration (54). A mouse model organism was utilized to evaluate skin inflammation with or without ultraviolet-B radiation exposure and with or without curcumin encapsulation in coconut oil. After 24 h of incubation, the experimental setup treated with encapsulated curcumin had less skin reddening than the control group (54). Moreover, inflammatory cytokine analyses and histology of the encapsulated curcumin-exposed skin revealed less skin cell damage and reduced inflammation (markers) compared to the control and non-encapsulated groups (54). Curcumin in nanocapsule or unencapsulated form efficiently restores host-microbe interactions and gut homeostasis (55).

### Curcumin nano formulation: Nanoparticles, nanocomposites, hydrogels

The nanoformulation of curcumin has been proven to have extensive therapeutic application (56). The most common nanoformulations of curcumin are solid lipid nanoparticles, nano-composite, nano-suspension, nanoparticles, liposomes, micelles, polymeric nanoparticles, and hydrogels (56). Liposomes are vesicular structures containing one or more phospholipid bilayers capable of transporting potential therapeutic molecules to target cells. Liposomes attach to the lipid membrane of the target cell and release their contents into the cytoplasm. Liposomes may encapsulate both hydrophilic and lipophilic compounds, ensuring maximum efficacy and safety in the delivery of target-specific drugs. Micelles are nanostructures having a hydrophilic outer layer and a lipophilic inside. The self-assembly of amphiphilic co-polymers forms them at a critical micellar concentration. The hydrophobic core is an effective drug transporter (56).

Nanosuspensions are insoluble water medications dispersed in aqueous solutions without a carrier. The drugs have a colloidal size distribution of fewer than 1 m and are stabilized using surfactants and other chemicals (57). The combination of small particle size (PS), a large surface area, and high thermodynamic energy (57) supports rapid drug dispersion. Solid lipid nanoparticles (SLNs) are colloidal lipid carriers (50–1000 nm) made of biological lipids that are biocompatible and biodegradable. Unlike liposomes, SLNs are rigid particles that can only be loaded with hydrophobic substances, such as curcumin. SLNs are distinguished by their high drug-loading capacity, robust stability, exceptional bio-compatibility, and enhanced bio-availability. Due to their hydrophobic nature, they are excellent nano-carriers for controlled release and site/cell-directed medicine delivery to reticuloendothelial cells (58).

Nanoemulsions (NEs) are kinetically stable, transparent, or translucent dispersions of oil, emulsifier, and water with particle sizes of less than 100 nm. In contrast, nano and micro-emulsions do not form spontaneously; their low surfactant concentration needs a large amount of energy to generate. Because they are emulsions, they permit the incorporation of both hydrophobic and hydrophilic therapies and the enhancement of hydrophobic medicines according to the size of their vesicles (59).

Nano-sized quantum dots, manganese phosphate nanoparticles, noble metals, carbon nanotubes, silica nanoparticles, and magnetic nanoparticles are inorganic. They feature unique, size-dependent physical properties, including optical and electrical effects, effective contrasting effects, and magnetism. In addition, they are resistant to microorganisms and possess excellent storage properties. Curcumin-based inorganic nanoparticles have the potential to be utilized in several vital bioapplications (60). Polymeric nanoparticles are colloids with a diameter of less than one millimeter composed of biodegradable or non-biodegradable natural or manufactured polymers. They are either nano-capsules (polymer encases the drug) or nano-spheres (where the medication is distributed throughout the polymer) (60). Liposomes have more reactivity, surface area, sensitivity, and stability than polymeric nanoparticles. Because of their high membrane permeability (due to their tiny size) and capacity to target specific organs, they interact with drug carriers (61).

Nanocomposites are non-homogenous materials made at the nanoscale level by combining polymers with inorganic solids. Their frameworks have been discovered to be more complex than micro composites. Individual property structure, composition, interfacial interactions, and components significantly impact them. The method of *in situ* growth, bio-polymer, and inorganic matrix polymerization is the most common way to create nanocomposites (62).

Hydrogels are three-dimensional (3D) polymeric structures that are extremely swollen, hydrophilic, and capable of absorbing huge volumes of water-insoluble compounds in water due to cross-linked polymers, enmeshment, or crystalline areas in their composition. Natural, synthetic, or hybrid polymers can be used to make hydrogels. Biopolymer-based hydrogels have attracted much attention as promising options for medicinal applications, including therapeutic delivery (63). Therefore, nanotechnology is a very effective tool to increase the limits of native curcumin to improve its therapeutic potential due to the presence of other key features such as high cellular uptake, biodistribution, dissociation rates, stability in serum, and sustained drug release at the target site.

### Structural analogs

Typically, curcumin analogs are divided into three groups: natural derivatives from turmeric, curcumin analogs from mother nature, and synthetic analogs (57) (Table 2). Curcumin, cyclocurcumin, bisdemethoxycurcumin, and demethoxycurcumin are curcuminoids that originate from turmeric. Bioactive compounds that occur naturally and possess structural similarity to curcumin are defined as curcumin analogs from mother nature. Synthetic analogs of curcumin compounds are produced by modifying the basic structure of curcumin using various chemical reactions. The major possibilities to modify curcumin structurally are making changes in the dike to functionality, aryl side chain, active methylene functionality, double bond, and metal complexes (64). Some of the synthetic analogs are EF24, (1E, 6Z) 1,7-bis(13-fluoro-9 -ethylcoumarin-8-yl)-5-hydroxy3-oxohepta-1,4,6-triene (65).

**TABLE 2:** List of natural derivatives and analogs of curcumin.

Scientific name/Common name	Compound name	Molecular formula/Molecular weight	PUBCHEM CID	References
*Curcuma longa* (Turmeric)	Curcumin	C_21_H_20_O_6_/368	969516	(66)
*Curcuma longa* (Turmeric)	Bisdemethoxycurcumin	C_19_H_16_O_4_/308	5315472	(67)
*Curcuma longa* (Turmeric)	Demethoxycurcumin	C_20_H_18_O_5_/356	5469424	(68)
*Curcuma longa* (Turmeric)	Cyclocurcumin	C_21_H_20_O_6_/368	69879809	(68)
*Zingiber officinale* (Ginger)	Dehydrozingerone	C_11_H_12_O_3_/192	5354238	(69)
*Zingiber officinale* (Ginger)	6-shogaol	C_17_H_24_O_3_/276	5281794	(69)
*Zingiber officinale* (Ginger)	6-paradol	C_17_H_26_O_3_/279	94378	(70)
*Zingiber cassumunar* (Ginger)	Cassumunin B	C_34_H_36_O_9_/588	10054109	(71)
*Zingiber cassumunar* (Ginger)	Cassumunin A	C_33_H_34_O_8_/558	10460395	(71)
*Zingiber officinale* (Ginger)	6-gingerol	C_17_H_26_O_4_/295	442793	(72)

### Liposomal curcumin

Curcumin is likely to be metabolized quickly with less photo-stability, making it an ineffective therapeutic agent in its natural form (73). According to several studies, solubilizing curcumin in a phospholipid bilayer and liposomes can increase its transport and bio-availability (74). It is well known that nanoparticles (liposomes, micelles) have been considered to improve curcumin’s intracellular and targeted drug delivery, which can cross inaccessible anatomical and physiological barriers. Thus, liposomes must be a successful method to increase the bioavailability and stability of curcumin (75). Major preparation methods to produce curcumin liposomes include an injection method, a reversed-phase evaporation method, a thin-film method, and a freeze-thawing method (76). Curcumin encapsulation, including liposomes for any cancer/disease, should enhance bioavailability compared to freely available curcumin. At the same time, it must be non-toxic to normal cells in the resulting environment. In this context, the synthesis of liposomes can be done using a solvent-free method (since solvents may be toxic). For instance, De Leo et al. (77) reported the encapsulation method of curcumin liposomes for drug delivery in pH-responsive polymers using an organic-free method. The pharmaco-dynamics and pharmaco-kinetics of liposomal curcumin were improved, resulting in higher anticancer and pharmacological activity. Especially, liposomal curcumin was prepared with various conjugates, including vitamin A, polyethylene glycol, hyaluronic acid, silica, and folic acid. Besides, the liposomal nanoparticles encapsulated with drug combinations could sensitize cancer cells in the OS cell line (human osteosarcoma cell line) (78). However, with the constant progress of various liposomes, curcumin liposomes should be more standardized for other diseases and cancer.

### Curcumin phospholipid complex

Several studies have implied the crucial roles of phospholipids in improving the therapeutic efficiency of small molecules for those with poor oral bioavailability (79). Generally, amphipathic phospholipid complexes act as bioactive components to pass them through the gastrointestinal cells to the blood (80). Theoretically, phospholipid complexes are appropriate strategies for any small bioactive molecule. The curcumin molecule is found to have a high affinity toward biological membranes and tends to penetrate them rapidly to form dimeric biological complexes. Despite being a phenolic and poorly soluble compound, curcumin can link with phospholipids (particularly phosphatidylcholine) by forming non-covalent adducts (80).

At last, the formation of these curcumin–phospholipid complexes could enhance the curcumin pharmacokinetics by stabilizing intestinal pH values and shielding curcumin in terms of retro-Claisen hydrolysis (36). Liu et al. (36) reported that a curcumin–phospholipid complex enhanced the oral bioavailability of curcumin compared with curcumin suspension to fivefold. In another study, *in vivo* absorption of a phospholipid–curcumin complex in Wistar rats showed that the bio-availability was significantly more enhanced than the kinetically free curcumin. In addition, pharmacokinetic study results revealed that a phospholipid–curcumin complex implied significantly high plasma concentrations and was found to be more stable when compared to natural curcumin (81). Various research findings suggest that the phospholipid–curcumin complex is one of the most precious methods for making curcumin more stable and improving its bioavailability (82).

## Biochemical transformation of curcumin

The biochemical transformation of curcumin may occur during the processing, storage, or preparation of food products through a variety of mechanisms, including oxidation, pH-induced instability, and photodegradation (82, 83).

### Oxidation

In the presence of oxygen, curcumin in phosphate buffer undergoes a rapid transformation into dicyclopentadiene, a major curcumin degradation product. This oxidation process was reported to be pH-dependent, with peak transformation at pH = 8, and catalyzed by cyclooxygenase-2 (COX-2) (39). Despite the rapid oxidation of curcumin into its degradation products, curcumin still exhibits its biological activities strongly; therefore, the activities may be because of the parent compound or degradation products. Nevertheless, a recent study comparing the anti-proliferative abilities of curcumin and its derivative, dicyclopentadiene, indicated that the derivative is significantly less active than the parent compound (83).

Furthermore, the co-addition of redox-active antioxidants (like ascorbate, TBHQ, and Trolox) dramatically increased curcumin stability, expanding its activities. With increasing chemical stability, curcumin efficacy significantly inhibits MC38 colon cancer cell proliferation. These data propose the biological impacts of curcumin are rather due to the parent compound, not the degradation. Furthermore, the co-administration of antioxidants increased curcumin concentration in plasma by approximately sixfold in an animal model (84). These results further supported the importance of stabilizing curcumin to increase its efficacies.

### pH-induced instability

Curcumin is also important as a pH indicator or biosensor, which can be applied in smart packaging. Curcumin can monitor food spoilage by indicating changes in food pH and packaging headspace and providing information about food quality. This curcumin molecule is due to its high sensitivity in detecting acid-base reactions with visible color changes in food to the naked eye, which the consumer can visualize through the packaging without the need to open it. The curcumin molecule at pH 3.0–7.0 usually has a yellow color. However, with the pH increase from 7 to 8 (alkaline conditions) there is a strong change from yellow to red. This change occurs because the phenolic hydroxyl group readily reacts with OH- to form the phenoxide anion and causes a color change (43, 85).

**FIGURE 2:**
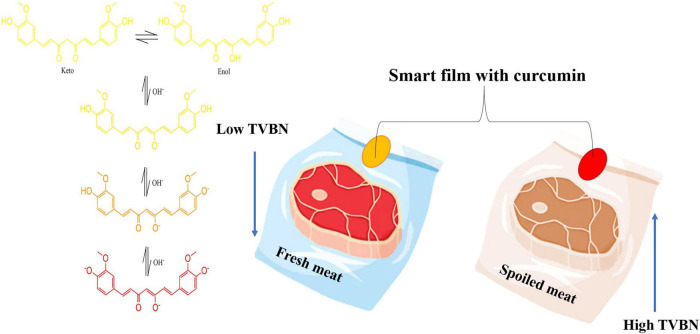
The effect of pH on curcumin color and its application in food shelf-life. TVBN, Total volatile base nitrogen.

Therefore, when adding curcumin to 0.1 M phosphate buffer at pH 7.2 and keeping at 37°C, more than 90% of the molecules rapidly degraded within 1 h, producing ferulic acid, vanillin, and feruloyl methane as its degradation products (Figure 2). HPLC analysis done by Nimiya et al. (84) showed that 80–90% of curcumin in phosphate buffer (pH 7.4) degraded post-12-min incubation. Another stability study showed that 80–90% of curcumin in phosphate buffer (pH 7.4) was degraded post-12-min incubation. Another stability study by Kharat et al. (86) at different pH values indicated that curcumin chemically degraded into its respective degradation products at alkaline pH values (pH ≥ 7.0), but crystallized, leading to a limitation of curcumin bioactivities at acidic pH values (pH < 7.0).

### Photodegradation

As curcumin undergoes degradation, the yellow color intensity decreases due to the formation of colorless products like vanillin, ferulic acid, and other small phenolic compounds. With exposure to sunlight, curcumin undergoes much faster degradation (87). High-performance thin layer chromatography analysis revealed that exposure to UV (254 nm) caused the degradation of curcumin into three products, whereas exposure to sunlight produced five degradation products (88). Few studies reported that singlet oxygen and superoxide were photogenerated by curcumin in biological systems, probably triggering curcumin phototoxicity. More studies on the mechanisms underlying curcumin photodegradation are required to understand the possible detrimental effects of curcumin phototoxicity (89).

### Biochemical transformation

Human bodies have been designed to metabolize and deactivate any foreign compounds entering their systems, including curcumin. So, when curcumin enters the body, it will be metabolized, degraded, and deactivated to prevent it from reacting with the body’s systems. A pharmacokinetic study of curcumin was conducted where healthy human volunteers were given a single oral dose of 10 and 12 g of curcumin. Then serum samples were evaluated for free curcumin, glucuronide conjugate, and sulfate conjugate. The data showed that free curcumin levels peaked in the plasma for 10 and 12 g doses at times of 3.29 ± 0.43 h and 6.77 ± 0.83 h, with peak concentrations of 2.3 ± 0.26 μg/mL and 1.73 ± 0.19 μg/mL respectively (90).

Most of the curcumin detected in the plasma was in its conjugate forms, where the ratio of glucuronide: to sulfate was 1.92:1 (90), indicating curcumin has poor metabolic stability. After compounds manage to be absorbed by the gastrointestinal tract inside our body, two types of metabolism processes occur to deactivate foreign compounds trying to enter our body. The absorbed compounds will first undergo phase 1 metabolism in the gut epithelium cells. After passing through the epithelium cells, some compounds will be pumped back into the lumen due to efflux. The remaining compounds will be transported through the blood circulation to the liver, where they undergo phase 2 metabolism (90).

#### Phase 1 metabolism

**FIGURE 3:**
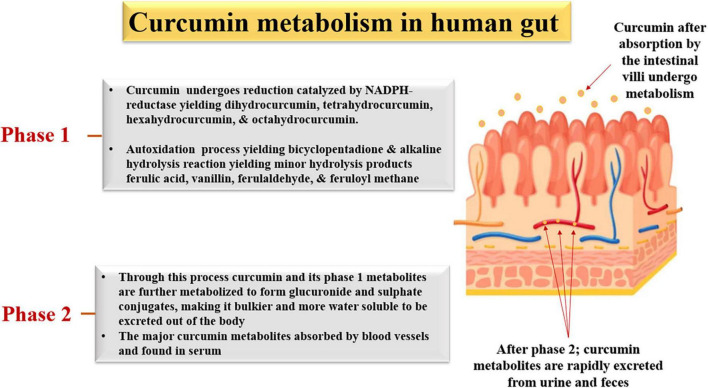
Phase I and Phase II metabolism of curcumin in epithelium cells.

As seen in Figure 3, during the phase 1 metabolism process in the epithelium cells, curcumin undergoes reduction catalyzed by NADPH-reductase, yielding dihydro curcumin, tetrahydro curcumin, hexahydro curcumin, and octa-hydro curcumin (phase 1 metabolites); autoxidation process yielding dicyclopentadiene and alkaline hydrolysis reaction yielding minor hydrolysis products ferulic acid, ferulaldehyde, vanillin, and feruloyl methane (91).

#### Phase 2 metabolism

This metabolism process includes conjugation reactions catalyzed by conjugative enzymes primarily found in the liver, kidney, and intestinal mucosa. Through this process, curcumin and its phase 1 metabolites are further metabolized, forming glucuronide and sulfate conjugates, making it bulkier and more water soluble. A recent study revealed that these conjugated products had fewer activations in HMVEC-dLy cells than curcumin due to their hydrophilicity (92).

Numerous animal and clinical studies have also found these metabolites to be the major curcumin form in serum. They are largely excreted out through feces and urine (93), indicating the deactivation of curcumin bioactivities through phase 2 metabolism and the rapid excretion of curcumin, leading to poor bioavailability of curcumin (93).

#### Metabolic regulation by gut microbiota

Curcumin metabolism pathways in the intestinal and liver cells have been intensively studied. Recent findings have shown the high importance of gut microorganisms in health, however, the fate of curcumin by intestinal microorganisms is largely unknown. Hassaninasab et al. (94) discovered a new metabolic pathway of curcumin involving a unique enzyme produced by the gut microorganism named CurA (NADPH-dependent curcumin or dihydro curcumin reductase). They were capable of isolating the curcumin-metabolizing microorganism from human feces and identifying it as *Escherichia coli*.

The 16S rRNA sequence of the *E. coli* H10407 strain, O55:H7 strain, CB9615, BW2952 strain, and K-12 strain DH10B sub-strain indicated their curcumin converting ability. CurA was responsible for converting curcumin through two reaction steps into dihydro curcumin and tetrahydro curcumin. However, the absence of further reduction products than tetrahydrocurcumin (THC) indicates that catalytic reduction by CurA is only effective for compounds with C = C double bonds (95). Future investigations are essential to understand the importance of gut microbiota in curcumin metabolic fate in the colon (96).

## Health benefits and biological impacts of curcumin

**FIGURE 4:**
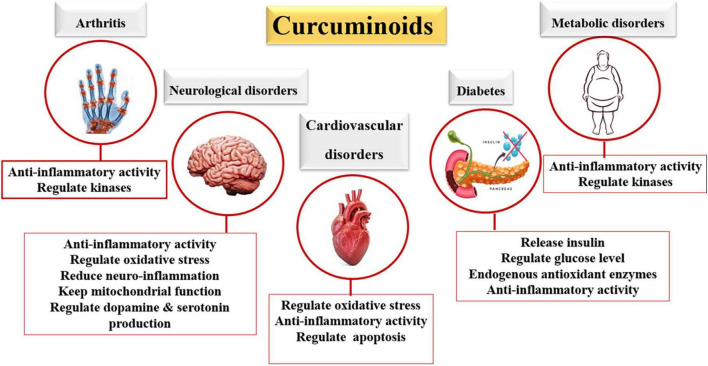
Health benefit of bioavailable curcumin.

As seen in Table 3 and Figure 4, curcumin has many different impacts on human health. Many researchers have studied the biological activities of curcumin in molecular, cellular, animal, and clinical studies to learn more about its contribution to health benefits. The obtained data showed that the actions of curcumin were involved in multiple pathways and mechanisms, demonstrating its beneficial effects. Curcumin has been studied for over three decades with numerous identified health benefits related to improvements in slowing the existence and progression of inflammatory disorders, cardiovascular disorders, and diabetes (97). For example, *in vivo* study when 50 patients with knee osteoarthritis were provided with a commercial compound containing curcumin (Theracurmin) (180 mg/day over 8 weeks) or a placebo, knee pain was significantly reduced by Theracurmin treatment when compared to the placebo (98). In addition, when another product containing curcumin (Algocur) was administered in pill form every 12 h for 5 or 10 days to rugby players suffering from osteo-muscular pain conditions, there were reductions in pain and improvements in physical function compared to the baseline condition (99). Curcumin has also been tested in cases with cardiovascular disorders, improving serum lipid levels (100).

**TABLE 3:** List of diseases cured by oral administration of bioavailable curcumin.

Disease		Dose/Duration	The cure effects	References
Cancer	Prostate	0.1 g/day for 6 months	Reduced serum PSA with the therapy of isoflavone and cur.	(109)
	Head and neck	–	Cur inhibited IKKβ kinase activity and IL-8 levels in head and neck cancer patients.	(110)
	Breast	6 g/day for 3 weeks	(1) The recommend dose for cur was 6000 mg/day for 3 weeks in combination with docetaxel. (2) Clinical responses were observed in patients for treatment of cur and docetaxel.	(111)
	Pancreatic	500 mg/day (cur), 5 mg (piperine) for 42 days	Decreased PhK (Phosphorylase kinase) activity in cur treated group.	(112)
		8 g/day for 4, 8 weeks	Cur oral administration is well tolerated with biological activity in pancreatic cancer, in spite of the limited absorption. Cur downregulated the cyclooxygenase-2, NF-κB, phosphorylated signal transducer in patients. The trial had positive outcomes with tumor regression and enhanced serum cytokines levels.	(113)
		8 g/day for 4 weeks	Cur had low compliance at high dose (8 g/day) Gemcitabine in combination with cur had therapeutic effect in advanced pancreatic cancer patients. Gemcitabine in combination with cur was safe and feasible in pancreatic cancer patients.	(113)
	Colorectal	0.44–2.2 g/day for 4 months	The oral bioavailability of cur was low and cur was metabolized by intestine. Metabolites were detected in the feces instead of the blood or urine. GST activity was decreased by 59% at a low dose (440 mg) instead of higher doses. Cur cause clinical benefit in patients of colon rectal cancer. Larger dose of cur clinical trial is merited.	(114)
		0.45–3.6 g/day for 4 months	Compounds and conjugates were detectable in plasma and urine after consumption of cur 3.6 g per day. Significantly decreased serum PGE2 levels at the highest dose.	(115)
		0.45, 1.8, 3.6 g/day for 7 days	Cur levels was pharmacologically efficacious by decreasing M 1 G levels instead of COX-2 protein levels in the colorectum with negligible cur distribution outside the gut at a dose of 3,600 mg/day. Cur was taken up by malignant and normal colorectal tissue. (3) Traces amount of cur was detected in the blood. The systemic availability of orally administered cur was low.	(116)
		2.4 g/day for month	Decreased ACF levels with the 4 g dose, while no decrease in 2 g dose.	(117)
		1.8 g/day for 10–30 days	Increased body weight, decreased serum TNF-α, induced p53, regulated apoptotic pathway of tumor cell.	(118)
Skin conditions	Psoriasis	4.5 g/day for 12 weeks	Cur with a dose of 4.5 g/day is safe and well-tolerated; Cur had treatment effect for psoriasis.	(119)
	Alzheimer’s disease	2–4 g/day for 24 weeks	Cur had a therapeutic effect on Alzheimer’s disease.	(120)
	Dejerine-Sottas Disease	1.5 g/day for 4 months	Cur had efficacy and safety on treatment of dejerine-sottas disease.	(121)
	Arterial diseases	0.5 g/day for 7 days	Increased HDL cholesterol (29%), decreased lipid peroxidase (33%), decreased total serum cholesterol (11.63%) were detected after cur administration.	(122)
Inflammatory diseases	Irritable bowel Syndrome	0.072–0.144 g/day for 8 weeks	Irritable bowel syndrome (IBS) prevalence was decreased; Abdominal pain was reduced.	(123)
	Ulcerative proctitis Crohn’s disease	0.350–0.550 g/day for 1–2 months	Cur had reduced inflammatory response effect in patients.	(124)
	Rheumatoid arthritis	0.5 g/day for 8 weeks	Cur had the treatment effect of active rheumatoid arthritis (RA).	(125)
	Chronic anterior Uveitis	0.125 g/day for 12 weeks	Side effect is lack; Recurrence rate was 86%.	(126)
	Recurrent anterior Uveitis	1.2 g/day for 12–18 months	More than 80% patients had reduced eye discomfort after treatment. Cur had therapeutic effect on eye relapsing diseases.	(126)
	Peptic ulcer	3 g/day for 4 weeks	Cur had the therapeutic effect of peptic ulcer.	(127)
	Inflammatory Orbital Pseudotumor	0.125 g/day for 6–22 months	Five patients completed the study, four patients recovered completely, the fifth had tumor related swelling. Cur had therapeutic effect on healing of peptic ulcer.	(128)
Metabolic diseases	Diabetes	0.6 g/day for 8 weeks	NCB-02 and atorvastatin can increased the endothelial function. NCB-02 had more therapeutic effect on endothelial dysfunction compared with atorvastatin can. The postprandial serum insulin levels were increased, plasma glucose levels or glycemic index was not increased after cur administration; Cur had the effect of insulin secretion.	(129)
	Diabetic microangiopathy	1 g/day for 4 weeks	Decrease in skin resting flux, edema score, increase in venoarteriolar response, PO 2 were observed.	(130)
	Lupus nephritis	0.5 g/day for 3 months	Proteinuria, hematuria, and systolic blood pressure were decreased.	(131)
	Diabetic nephropathy	1.5 g/day for 2 months	TGF-β and IL-8 serum level and urinary protein excretion were decreased after cur administration. No adverse effect was observed.	(132)
	Renal transplantation	0.480–0.960 g/day for a month	Enhanced effect in cadaveric renal transplantation.	(133)
Others	Safety trials Phase I	0.50–12 g/day for 3 months	Cur is not toxic up to the dose of 8,000 mg/day for the treatment of 3 months. Cur was not absorpted by gastrointestinal completely with low Cmax (1.77 ± 1.87 μM/ml at the dose of 8,000 mg). Curmin had a biologic effect in cancer treatment.	(112)
	β-thalassemia	0.5 g/day for 12 months	Oxidative stress was increased in β-thalassemia/Hb E patients after cur administration.	(134)
	Alcohol intoxication	0.03 g for single dose	The bioavailability of THERACURMIN was higher than cur powder for the treatment of human disorders.	(135)
	Atherosclerosis	0.020 g/day for 28 days	Increased ApoA and HDL, decreased apoB and LDL after cur administration.	(136)
	Respiratory contraction	3 g/day for 4 weeks	Reduced infections after administration of lactoferrin and cur (LC).	(137)

Intake of curcumin extract (650 mg three times daily for 12 weeks) in patients with metabolic syndrome exhibited a lowering effect on the low-density lipoprotein cholesterol and an elevating one on the high-density lipoprotein cholesterol compared to placebo (101). The use of curcumin in treating type 2 diabetes was also intriguing. At a dose of 0.2/100 g or 1.0 g/100 g, a curcumin extract was fed to type 2 diabetic KK-A mice for month, compared to a basal control diet. The supplemented groups could resist the increased blood glucose observed in the control group, referring to turmeric as a functional food in type 2 diabetes mellitus (102). Similar findings have been noticed in human subjects. For example, *in vivo* ingestion of 6 g of *C. longa* elevated postprandial serum insulin levels at the 30- and 60-min intervals during an oral glucose tolerance test (19). Altogether, these studies, and others, showed that curcumin may be effective in controlling circulating blood glucose concentrations and has the potential to slow the progression of type 2 diabetes (15, 70–72). Turmeric is a safe food that healthy people can eat in moderation without getting sick (16).

Even if all the nutrients and substances in nature are beneficial for human health, they can become harmful when consumed in excess. There is a maximum level that should not be exceeded. Different investigations have referred to the safety of polyphenols at high doses (up to 8 g per day), but a clear dose has not yet been determined or decided. The Oxygen Radical Absorption Capacity (ORAC) value, which shows the antioxidant capacity of foods, is 44.776 in turmeric. With this value, turmeric ranks first in the list of spices with the highest antioxidant capacity. ORAC is a scale called free radicals in our body that indicates the absorption of substances that cause many diseases, especially cancer. That is, it is used for nutrients that indicate the absorption value. A food with a high ORAC value is more antioxidants, protects against cancer, and slows aging (16).

For adults, turmeric can be used as capsules, liquid essence, and tinctures containing powder. The cut root of turmeric can be consumed daily at 1.5–3 g, and the dried powdered turmeric root at 1–3 g. A standard powder (curcumin) can be taken thrice daily at 400–600 mg. Liquid extract (1:1) is recommended for daily consumption at 30–90 drops, one dose in the morning and one in the evening (1 part is 5 ml). Fresh turmeric can be stored in a cool, dry place for several weeks (103).

Curcumin can be used in salads, rice, and meat dishes to increase the flavor and consistency of food, giving it an attractive yellow color. It can be added to the dishes by mixing them with honey. It is also added to fish soup, cold cuts, and various vegetable dishes as a seasoning. It is used in the Spanish’s famous “paella” dish and the “curry” sauce of the Indians. Turmeric can also be used as a tea, which is very popular in Asian countries, especially Japan (103). Curcuma, the active ingredient of turmeric, prevents cancer and Alzheimer’s, protects from heavy metals, heals the liver, and is also a powerful antioxidant. However, for this effect of turmeric, the doses that will provide the same therapeutic effect at different times should be correct. Curcuma is great when it is pure and in small quantities, but its taste is bitter and less enjoyable when taken in larger doses (104).

Scientific data reveal that curcumin, is a difficult substance to absorb, and accordingly, it has poor bioavailability as well. Curcumin is absorbed very little in the human body when taken alone and is rapidly excreted from the intestines. For this reason, many studies have been conducted on the substances that will increase the bioavailability of curcumin, and it has been recommended that the piperine contained in black pepper can improve the uptake of curcumin by 2,000% (20 times). The vast metabolism of turmeric in the hepatic tissues and intestinal walls increased its bioavailability, which improved through piperine, which increases the absorption of all nutrients (104).

If such an improvement is not used, very little curcumin is absorbed, and even doses up to 4,000 mg can be completely inactive. Scientific research also shows that piperine, the active ingredient of black pepper, increases the absorption of substances that are difficult to absorb. Taking these two substances with an oil rich in unsaturated fatty acids further strengthens this benefit (74). With the extensive use of natural flavors in the food industry, the attention to natural antioxidants in plants worldwide is elevating daily. Since natural ingredients are used a lot in the food industry, people worldwide are becoming increasingly interested in natural antioxidants in herbs (105).

India has significant potential in terms of the production and export of some herbs and spices. Knowing these herbs and spices’ antioxidant and antimicrobial activities will make an essential contribution to extending the shelf life of food products (105, 106). Foods rich in polyunsaturated fatty acids are exposed to oxidative deterioration, which limits the shelf life of food products and causes quality loss. In industrial processes, synthetic antioxidants are mainly employed to prolong the preservation of nutrients. Many researchers state that some synthetic antioxidants used in food processing are carcinogenic and teratogenic to living organisms. Thus, consumers generally prefer natural antioxidants over synthetic ones (106).

Indian aromatic herbs used as additives to increase the sensorial properties of nutrients such as smell, and taste have become increasingly important. The antioxidant effect of phenolic materials found in the composition of these plants is based on their biochemical activities, e.g., cleaning free radicals, compounding metal ions, and preventing the formation of single oxygen. Some herbs and spices have been proven to have more antioxidant capacity than synthetic antioxidants (107). Because of the specific flavors and aromas, as well as the antimicrobial and antioxidant properties, herbs and spices have a broad bioactivity profile and can be utilized as alternatives in the food industry. Since, the prevention of lipid oxidation in foods is crucial for the producer and consumer, turmeric represents a good chose choice, being a strong natural antioxidant (108).

### Curcumin’s antioxidant activity

The accumulation of reactive oxygen species (ROS) in cells has been tied to many disease cases, including the damage of the nucleic acids and DNA, potentially causing mutations and leading to tumor formation. ROS are free radicals involving superoxide hydroxyl radicals, anion radicals, hydrogen peroxide, and singlet oxygen. The adverse effects of ROS can be mitigated by natural antioxidants. The experiments on the *in vitro* stability and antioxidant capacity showed that when curcumin is embedded in the O/W SFME, its storage stability, light stability, thermal stability and antioxidant activity are significantly improved. This research is of great significance for the practical application of curcumin and other biomolecules (138). Also, in this study, glycated soy β-conglycinin (β-CG) stabilized curcumin (Cur) composites which were fabricated by a unique reversible self-assembly character of β-conglycinin-dextran conjugates (β-CG-DEX). Intrinsic fluorescence and far-UV CD spectra revealed that glycation did not affect the self-assembly property of β-CG in the pH-shifting treatment. The structure of β-CG-DEX could be unfolded at pH 12.0 and reassembled during acidification (from pH 12.0 to 7.0). Meanwhile, β-CG-DEX-3d, which was incubated at 60°C for 3 days, exhibited a high loading capacity (123.4 mg/g) for curcumin, which far exceeds that (74.90 mg/g) of β-CG-Cur. Moreover, the reassembled β-CG-DEX-3d-Cur showed eminent antioxidant activity of approximately 1.5 times higher than that of free curcumin. During the simulated gastrointestinal condition, compared with β-CG-Cur, β-CG-DEX-3d-Cur nanoparticles showed a more stable and sustained release of curcumin. Thus, β-CG-DEX has immense potential to become a new delivery carrier for hydrophobic food components by means of a self-assembly strategy (139). Turmeric is beneficial for the liver. It strengthens the liver and helps remove toxins from the liver (140–142).

### Anti-microbial impacts of curcumin

**FIGURE 5:**
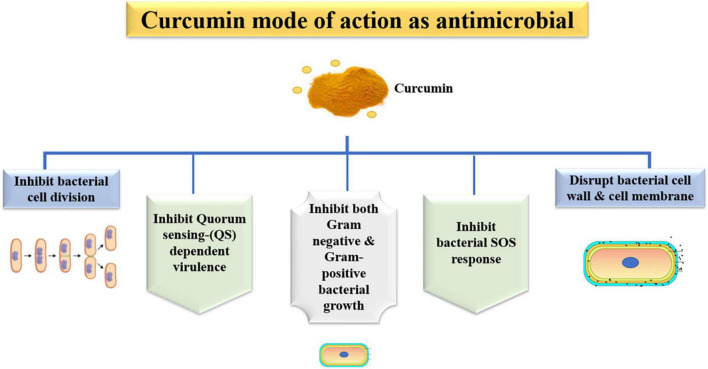
Antimicrobial mechanism of bioavailable curcumin against pathogenic microorganisms.

**FIGURE 6:**
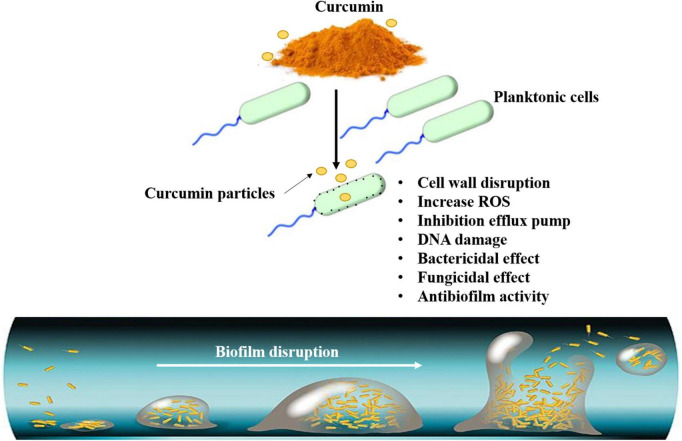
Antibiofilm mechanism of bioavailable curcumin against pathogenic microorganisms.

The natural components from plants are considerable antimicrobial agents may that can substitute the current antibiotics, which are facing increasingly evolving resistance by microorganisms (Figure 5) (143–155). In an *in vitro* study, curcumin inhibited the growth of antibiotic-resistant *Pseudomonas aeruginosa*, *Acinetobacter baumannii*, and *Klebsiella pneumoniae* isolated from burn wound infections and showed synergism with meropenem (156). On gastrointestinal bacteria of human and bovine origin, Cur inhibited Firmicutes [*Clostridioides difficile* and *Acetoanaerobium* (*Clostridium*) *sticklandii*] but did not affect Bacteroidetes (*Bacteroides fragilis* and *Prevotella bryantii*) (157). Cur was conjugated to triphenyl phosphonium resulting in a compound named Mitocurcumin, which inhibited the growth of *Bacillus subtilis*, *E. coli*, *Staphylococcus carnosus*, and *Mycobacterium smegmatis*, and induced morphological changes in *B. subtilis* (158). Seventeen synthesized monocarbonyl curcuminoids showed high antibacterial activity against MSSA and MRSA and moderate activity against *E. coli* (158). The four most effective curcuminoids were bacteriostatic at low concentrations and bactericidal at high concentrations against MRSA, which showed membrane damage. In an *ex vivo* mammalian co-culture infection model, two curcuminoids decreased the viability of MSSA internalized in the fibroblasts (158). One of 13 synthesized curcuminoids, 3,3’-dihydroxycurcumin, showed antibacterial activity against *S. aureus*, *B. subtilis*, *Enterococcus faecalis*, and *Mycobacterium tuberculosis*, and produced membrane damage on *B. subtilis* (159). Nonetheless, all the synthesized curcuminoids were not effective against Gram-negative species (*P. aeruginosa* and *E. coli*). Cur analogs (monocurcuminoids, MC) were synthesized and showed higher, lower, or similar antimicrobial activity than Cur against *Aeromonas hydrophila*, *E. coli*, *E. faecalis*, *K. pneumoniae*, *P. aeruginosa*, *S. aureus*, and the yeast *Candida albicans*. Two MC and turmeric powder presented synergism against *A. hydrophila*, *P. aeruginosa*, and *C. albicans*. When aPDT was performed with UV light, two MC-mediated aPDT decreased the growth of *E. faecalis*, *E. coli*, and *S. aureus*, while aPDT with another MC and Cur increased the growth of *A. hydrophila*, *E. faecalis*, *S. aureus*, *C. albicans*, and *P. aeruginosa* (160). Cur was more effective than other natural biomolecules (quercetin and resveratrol) in inhibiting the growth of *E. faecalis* in spermatozoa from rabbits, but less effective than antibiotics (161). Cur-mediated aPDT also reduced the viability of *E. faecalis* biofilms grown in bovine bone cavities for 14 days by 1.92 log_10_ (162). The aPDT and the combination of a nanobubble solution and ultrasound reduced the viability of the aquatic pathogens *A. hydrophila* and *Vibrio parahaemolyticus* (163). The curcumin mode of action as an antimicrobial agent is summarized in Figures 5, 6.

### Anti-inflammatory impacts of curcumin

**FIGURE 7:**
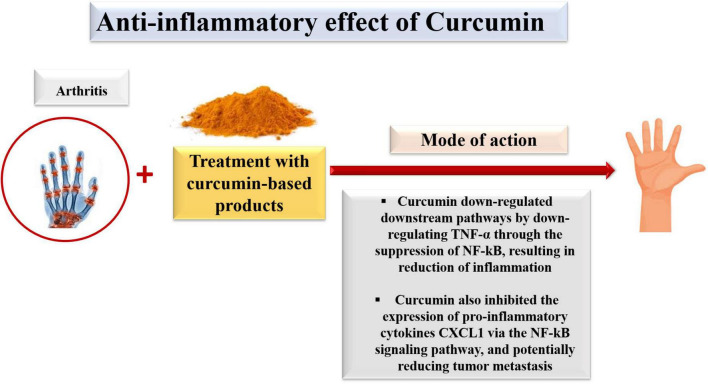
Anti-inflammatory mechanism of bioavailable curcumin.

Inflammation is the reaction of the body’s immune system associated with many diseases (Figure 7) and is a significant risk factor for tumor progression, such as tumor development and metastasis (164). In mammalians, the nuclear factor kappa B (NFkB) pathway, which plays a significant action in intracellular activities, can be activated by various agents, including pro-inflammatory cytokines, e.g., tumor necrosis factor-alpha (TNF-α). Once activated, it will, in turn, activate the downstream inflammatory pathways such as cyclooxygenase (COX-2), lipoxygenase (LOX), and inducible nitric oxide synthase (iNOS). Curcumin has been shown to down-regulate these downstream pathways by suppressing NF-kB and thus decreasing inflammation. Curcumin, a natural compound, inhibits the expression of pro-inflammatory cytokines such as CXCL1 through the NF-kB signaling pathway, potentially reducing tumor metastasis (148, 152, 153, 165).

Zhang (166) found that Cur loaded tetrahedral framework nucleic acids (Cur-TFNAs) were synthesized to deliver Cur. Compared with free Cur, Cur-TFNAs exhibit a preferable drug stability, good biocompatibility (CCK-8 assay), ease of uptake (immunofluorescence), and higher tissue utilization (*in vivo* biodistribution). Most importantly, Cur-TFNAs present better anti-inflammatory effect than free Cur both *in vivo* and *in vitro* experiments through the determination of inflammation-related cytokines expression. Therefore, Cur-TFNAs have great prospects for the prevention of gout and similar inflammatory diseases.

### Immune stimulant impact of curcumin

An *in vivo* study evaluated the impacts of turmeric as a dietary supply for the ornamental fish Green Terror on the development and performance, survival rate, and blood indices. A total of 144 samples with an average weight of 1.53 ± 0.22 (g) were collected, and the hypothesis was investigated with four isocaloric and iso-nitrogenous diets containing 0.1, 0.2, and 0.3% of turmeric powder. Over a 100 days, the fish were subjected to biometrics every 20 days. At the end of the experiment, blood samples were tested. Findings revealed that the fish fed with 0.3% turmeric powder showed better growth performance, feed conversion ratio (FCR), condition factor, and survival rate specification, but RBC, PCV, hemoglobin, and MCHC were not elevated significantly (*p* > 0.05) contrasted to the control fish (*p* > 0.05) (167).

In another study, 180 Nile tilapia fish were applied in 3 monthly growth trials to observe the impact of turmeric on growing tilapia. It was noticed that 0.50% turmeric might enhance growth performance and significantly keep fish versus *P. fluorescens* challenge (168). The impact of turmeric on the immune response of fish *Labeo rohita* has been judged an effective action (169). Also, *in vivo* study reported that curcumin had a protective impact on Bloch tissue and elevated the growth performance (170). In this regard, the consumption of turmeric by fantail guppy (*Poecilia reticulate*) caused the reduction of FCR and improved growth performance (171). Sand Goby (*Oxyeleotris marmoratus*) also showed a positive reaction to the consumption of turmeric powder by increments in the secretions of amylase, lipase, trypsin, and chemo-trypsin (172).

The effectiveness of turmeric powder in combination with ginger and garlic on the immunity response of *Labeorohita* to *A. hydrophila* and white shrimp (*Litopenaeus vannamei* boone) has been evaluated by Chowdhury et al. (173). The impact of turmeric on blood and immunological status of *Mugil cephalus* vaccinated with *A. hydrophila* bacterium has been assessed (174). The immune response of Japanese flounder and Nile Tilapia fingerlings was enhanced when fed a diet supplemented with curcumin (175, 176).

In a different study, the effect of dietary curcumin on *Cyprinus carpio*, Rohmah et al. (177) investigated the potentials of dietary curcumin and resveratrol on blood biochemistry, immune responses and resistance to the toxicity of the pesticide, abamectin. Common carps (540) (30.78 ± 0.17 g) were randomly distributed into 18 tanks (30 fish per tank), as six experimental groups (T1: non-supplemented and on-exposed fish, T2: 300 mg/kg curcumin, T3: 300 mg/kg resveratrol, T4: 12.5% LC_50_ of abamectin, T5: 300 mg/kg curcumin + 12.5% LC_50_ of abamectin, T6: 300 mg/kg resveratrol + 12.5% LC_50_ of abamectin). Use of 300 mg/kg resveratrol in the diet of non-abamectin exposed fish improved the growth performance (*P* < 0.05), while such effects were not observed for curcumin (*P* > 0.05). There were no differences in the final weight (FW), FCR and weight gain (WG) between control and fish of the treatments, resveratrol + abamectin and curcumin + abamectin (*P* < 0.05). The immune components in blood [lysozyme, complement activity, total immunoglobulin (total Ig), protease, myeloperoxidase (MPO), nitro-blue-tetrazolium (NBT), peroxidase, albumin] and mucus [acid phosphatase (ACP), alkaline phosphatase (ALP), esterase, antiprotease)] and antioxidant enzymes [(superoxide dismutase (SOD), glutathione peroxidase (GPx)] exhibited various change patterns compared to the control group, however, these components were almost all higher in fish supplemented with curcumin and resveratrol in an abamectin-free medium than in control and other groups (*P* < 0.05) (177). In most cases, the levels of immune and antioxidant components in the control did not show significant difference with the treatments, resveratrol + abamectin and curcumin + abamectin (*P* > 0.05). Abamectin induced oxidative stress in fish, as the malondialdehyde (MDA) levels significantly increased in the exposed fish compared to non-exposed groups (*P* < 0.05) (177). It appears that neither curcumin nor resveratrol were as effective in preventing oxidative stress, because MDA levels were higher in exposed fish (abamectin, curcumin + abamectin, resveratrol + abamectin) than in control and non-exposed individuals (*P* < 0.05). Curcumin and resveratrol also showed protective effects on liver, since the levels of liver metabolic enzymes [aspartate transaminase (AST), ALP, lactate dehydrogenase (LDH)] were lower in the supplemented fish in an abamectin-free medium than in control (*P* < 0.05) (177). Curcumin and resveratrol also mitigated the stress responses in the exposed fish, as cortisol and glucose levels showed significant decreases in the supplemented fish (*P* < 0.05). In conclusion, this study revealed that abamectin can depress the growth and immunity in the common carp. Although, both resveratrol and curcumin were mitigated the toxic effects of abamectin, it seems that resveratrol be more effective than curcumin (177).

### Curcumin and Alzheimer

Progressive degeneration and dysfunction of the nervous system because of oxidative stress, aggregations of misfolded proteins, and neuroinflammation are the key pathological features of neurodegenerative diseases. Alzheimer’s disease (AD) is a chronic neurodegenerative disorder driven by uncontrolled extracellular deposition of β-amyloid (Aβ) in the amyloid plaques and intracellular accumulation of hyperphosphorylated tau protein. Many different signaling pathways, such as Wnt/β-catenin, Notch, ROS/JNK, and PI3K/Akt/mTOR are involved in Alzheimer’s disease and crosstalk between themselves. Curcumin is a hydrophobic polyphenol with noticeable neuroprotective and anti-inflammatory effects that can cross the blood-brain barrier. Therefore, it is widely studied for the alleviation of inflammatory and neurological disorders. However, the clinical application of curcumin is limited due to its low aqueous solubility and bioavailability because it has difficulty permeating the blood–brain barrier (BBB), it must be encapsulated by a drug carrier. To increase curcumin’s permeability across the blood–brain barrier, it was encapsulated and conjugated with different agents. The studies indicated that lipid-based carriers and poly lactic-co-glycolic acid (PLGA) increased its organ distribution tremendously (Table 3). The functionalization of curcumin with metallic nanoparticles also enhanced the uptake of curcumin in the brain. Furthermore, the conjugation of carriers with targeting agents, such as Tet-1 peptide, transferrin, lactoferrin, and chitosan increased the blood–brain barrier permeability of curcumin. However, research on the mechanism of curcumin with a delivery vehicle is limited. Therefore recently, nano-based curcumin delivery systems are developed to overcome these limitations effectively (178).

Also, some of the most frequently studied are lipid nanocarriers, liposomes, micelles and PLGA. These carriers are further conjugated with brain-targeting agents such as lactoferrin and transferrin. Curcumin was investigated heavily as a treatment for AD. It stimulated neurogenesis via the Notch and Wnt/β-catenin pathways, diminished the secretion of proinflammatory cytokines, and led to the deactivation of GSK-3β, which in turn reduced Aβ production and the buildup of plaques by downregulating the ROS/JNK pathway (178). Furthermore, downregulation of NF-κB signaling led to GSK3β-mediated inhibition of *BACE1*, which ultimately reduced *A*β plaques. However, the exact mechanism by which curcumin regulated these processes is still unknown. It would be beneficial to study the important signaling pathways after curcumin is encapsulated with nanocarriers to see if the action mechanism of curcumin is sustained (178). Overall, curcumin is a very promising antioxidant for the treatment of AD, and the use of carriers and targeting agents is very effective for enhancing delivery to the brain (179).

Furthermore, Campisi et al. (180) assessed the effect of the systemic administration of SLNs to facilitate Cur delivery on TG2 isoform expression levels in Wild Type (WT) and in TgCRND8 (Tg) mice. An experimental model of AD, which expresses two mutated human amyloid precursor protein (APP) genes, was used. Behavioral studies were also performed to evaluate the improvement of cognitive performance and memory function induced by all treatments (180). The expression levels of Bcl-2, Cyclin-D_1_, and caspase-3 cleavage were evaluated as well. The systemic administration of SLNs-CUR, both in WT and in Tg mice, allows one to differently modulate TG2 isoforms, which act either on apoptotic pathway activation or on the ability of the protein to repair cellular damage in the brains of Tg mice. In this study, it was also suggested that SLNs-CUR could be an innovative tool for the treatment of AD (180).

### Anti-cancer impacts of curcumin

**FIGURE 8:**
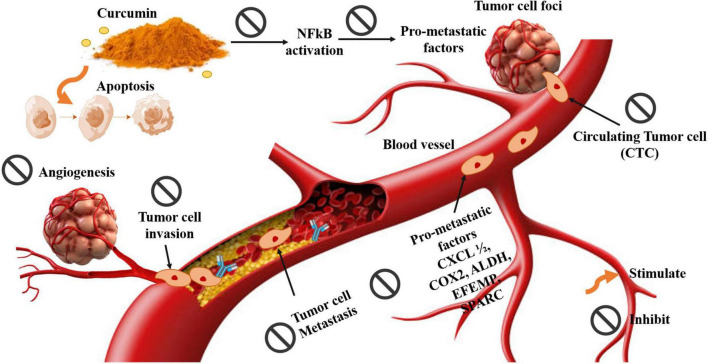
Anticancer mechanism of bioavailable curcumin against numerous types of cancer.

Many investigations have clarified that curcumin has potent anti-cancer impacts via suppressions of angiogenesis formation of new blood vessels from the preexisting vessels (181). There are multiple steps involved in angiogenesis, including activation, proliferation, invasion, and migration of the endothelial cells (182). Curcumin has been shown to prevent angiogenesis via multiple suppression of these steps in different cancers. Moreover, studies showed that curcumin also inhibited lymphangiogenesis, the formation of new lymphatic vessels, which has a serious function in cancer metastasis, *in vivo* through suppression of VEGF Receptor signaling (141, 155, 183). Indian diets contain a lot of fried foods, which also contribute to gastrointestinal tract cancers due to the cooking process, probably forming carcinogenic or mutagenic heterocyclic amines (HA). Some animal studies showed that feeding the mice with typical Indian dishes such as deep-fried vegetables showed a 20% increase in gastric carcinoma. However, despite all this, gastric tumor incidence rates are rated as moderate to low in India, in contrast to other countries. High use of natural compounds, e.g., turmeric, may explain why they protect against the cancer-causing bacterium *Helicobacter pylori*, a common cause of stomach tumors (184–186). The following some examples of tumors that enhanced by curcumin or curcumin composites and the anticancer mechanism of bioavailable curcumin illustrated in Figure 8.

#### Colon cancer

One of the significant issues of the anti-cancer effects of phytochemicals, bioactive compounds from foods, and other plants, is that the effective dosages of the phytochemicals are too high to be obtained by oral intake, particularly by food intake. In the study of Aromokeye and Si (187) a combination of curcumin and luteolin, two phytochemicals from food, at lower concentrations showed a higher inhibitory effect on colon cancer growth and investigated possible molecular mechanisms of this anti-colon cancer effect. By pairwise combination screening, the combination of curcumin (Cur) at 15 μM and luteolin (LUT) at 30 μM (C15L30) synergistically suppressed the proliferation of human colon cancer CL-188 cells, but the individual chemicals had a little inhibitory effect at the selected concentrations. This result was also confirmed in other colon cancer DLD-1cells, suggesting that this synergistic inhibitory effect of C15L30 applies to different colon cancer cells. The combination C15L30 synergistically suppressed the wound closure (wound healing assay) in CL-188 cells. The combination of Cur and LUT (at 20 mg/kg/day and 10 mg/kg/day, respectively, IP injection, 5 days for 2 weeks) synergistically suppressed tumor growth in CL-188 cell-derived xenograft mice (187). Western blot results showed that protein levels of Notch1 and TGF-β were synergistically reduced by the combination, both in CL-188 cells and xenograft tumors. Tumor pathological analysis revealed that combined Cur and LUT synergistically increased necrosis, but the individual treatment with Cur and LUT had no significant effect on tumor necrosis. Therefore, combined curcumin and luteolin synergically inhibited colon cancer development by suppressing cell proliferation, necrosis, and migration associated with Notch1 and TGF-β pathways. Colon cancer may be prevented/treated by consuming foods having high levels of luteolin and curcumin in humans (187).

Additionally, curcumin reverses doxorubicin resistance in colon cancer cells at the metabolic level, where Zhang et al. (188) examined the MDR reversal capability of Cur in drug sensitive-(SW620) and resistant-(SW620/Ad300) colon cancer cells, and elucidated the underlying molecular mechanisms at the metabolic level. It was found that Cur reversed P-gp-mediated resistance in SW620/Ad300 cells by enhancing the Dox-induced cytotoxicity and apoptosis. Further mechanistic studies indicated that Cur inhibited the ATP-dependent transport activity of P-gp, thereby increasing the intra-cellular accumulation of Dox in drug-resistant cells. Metabolomics analysis based on UPLC-MS/MS showed that the MDR phenomenon in SW620/Ad300 cells was closely correlated with the upregulation of spermine and spermidine synthesis and D-glutamine metabolism. Cur significantly inhibited the biosynthesis of spermine and spermidine by decreasing the expression of ornithine decarboxylase (ODC) and suppressed D-glutamine metabolism, which in turn decreased the anti-oxidative stress ability and P-gp transport activity of SW620/Ad300 cells, eventually reversed MDR. These findings indicated the MDR reversal activity and the related mechanism of action of Cur, suggesting that Cur could be a promising MDR reversal agent for cancer treatment.

#### Lung cancer

The application of traditional chemotherapy drugs for lung cancer has obvious limitations, such as toxic side effects, uncontrolled drug-release, poor bioavailability, and drug-resistance. Thus, to address the limitations of free drugs and improve treatment effects, novel T7 peptide-modified nanoparticles (T7-CMCS-BAPE, CBT) was developed based on carboxymethyl chitosan (CMCS), which is capable of targeted binding to the transferrin receptor (TfR) expressed on lung cancer cells and precisely regulating drug-release according to the pH value and ROS level. The results showed that the drug-loading content of docetaxel (DTX) and curcumin was approximately 7.82 and 6.48%, respectively. Good biosafety was obtained even when the concentration was as high as 500 μg/mL. More importantly, the T7-CMCS-BAPE-DTX/CUR (CBT-DC) complexes exhibited better *in vitro* and *in vivo* anti-tumor effects than DTX monotherapy and other nanocarriers loaded with DTX and Cur alone. Furthermore, CBT-DC ameliorated the immunosuppressive micro-environment to promote the inhibition of tumor growth. Collectively, the current findings help lay the foundation for combinatorial lung cancer treatment (189). Also, curcumin found to induce ferroptosis in non-small-cell lung cancer via activating autophagy, through inhibiting tumor growth and cell proliferation, but promoted cell death. Characteristic changes of ferroptosis were observed in curcumin group, including iron overload, GSH depletion and lipid peroxidation. Meanwhile, the protein level of ACSL4 was higher and the levels of SLC7A11 and GPX4 were lower in curcumin group than that in control group. Incubation of ferroptosis inhibitors ferrostatin-1 (Fer-1) or knockdown of iron-responsive element-binding protein 2 (IREB2) notably weakened curcumin-induced anti-tumor effect and ferroptosis in A549 and H1299 cells. Further investigation suggested that curcumin induced mitochondrial membrane rupture and mitochondrial cristae decrease, increased autolysosome, increased the level of Beclin1 and LC3, and decreased the level of P62. Curcumin-induced autophagy and subsequent ferroptosis were both alleviated with autophagy inhibitor chloroquine (CQ) or siBeclin1 (190).

#### Prostate cancer

Prostate cancer is the most widespread tumor in the U.S.A. The malignancy of this cancer is due to its ability to evolve into its refractory hormone stage. A major challenge in the clinical management of prostate cancer (PC) is to inhibit tumor growth and prevent metastatic spreading. In recent years, considerable efforts have been made to discover new compounds useful for PC therapy, and promising advances in this field were reached. Drugs currently used in PC therapy frequently induce resistance and PC progresses toward metastatic castration-resistant forms (mCRPC), making it virtually incurable. Curcumin, a commercially available nutritional supplement, represents an attractive therapeutic agent for mCRPC patients. The effects of chemotherapeutic drugs such as docetaxel, paclitaxel, and cisplatin, to curcumin, was compared on two PC cell lines displaying a different metastatic potential: DU145 (moderate metastatic potential) and PC-3 (high metastatic potential). The results revealed a dose-dependent reduction of DU145 and PC-3 cell viability upon treatment with curcumin similar to chemotherapeutic agents (paclitaxel, cisplatin, and docetaxel). Furthermore, the EGFR-mediated signaling effects on ERK activation in DU145 and PC-3 cells was explored (191). Results showed that DU145 and PC-3 cells overexpress EGFR, and the treatment with chemotherapeutic agents or curcumin reduced EGFR expression levels and ERK activation. Finally, chemotherapeutic agents and curcumin reduced the size of DU145 and PC-3 spheroids and have the potential to induce apoptosis and also in Matrigel. In conclusion, despite different studies being carried out to identify the potential synergistic curcumin combinations with chemopreventive/therapeutic efficacy for inhibiting PC growth, the results showed the ability of curcumin used alone, or in combinatorial approaches, to impair the size and the viability of PC-derived spheroids (191). Additionally, curcumin has considerable role in inhibiting PC progression by regulating the miR-30a-5p/PCLAF axis, where impede the proliferation, migration and invasion of PC cells, and promote their apoptosis in a time- and dose-dependent manner. Curcumin enhanced miR-30a-5p expression and inhibited PCLAF expression; furthermore, there was a negative correlation between miR-30a-5p and PCLAF expression in PC tissues. In addition, transfection of miR-30a-5p inhibitors partially reversed the function of curcumin on cell proliferation, migration, invasion and apoptosis. Overall, curcumin suppressed the malignant biological behaviors of PC cells by regulating the miR-30a-5p/PCLAF axis (192).

#### Pancreatic cancer

Pancreatic carcinoma is a malignant tumor with a high fatality rate, and the increased resistance of pancreatic carcinoma to chemotherapy has become a difficult problem in clinical practice. Hence, it is imperative to develop an effective treatment for pancreatic cancer. Sestrins are a class of stress-induced proteins that have antioxidation functions, regulating cell growth and metabolism. Curcumin is a natural pigment isolated from turmeric. Several studies have also suggested that this molecule has multiple pharmacological effects, such as anti-inflammatory, antioxidant, and antitumor effects. However, there are insufficient studies on curcumin cooperating with the sestrin family to inhibit tumors, and the mechanism is still unclear. A study carried out (193) observed the potential anticancer effects of curcumin combined with the sestrin family on pancreatic carcinoma and probe its possible molecular mechanisms. The results revealed that curcumin cooperated with sestrin2 to significantly suppress pancreatic cancer. In addition, sestrin2 cooperated with curcumin to inhibit pancreatic cancer by specifically targeting Nrf2/Keap1/HO-1/NQO-1. It is concluded that curcumin-mediated synergistic targeting of sestrin2 is a potentially valuable treatment for pancreatic cancer (193). In another study, a solid phase approach was proposed for the combinatorial synthesis of a mini library of the mimics of curcumin in good purity and yield. The non-effective findings in pancreatic cancer cells switched to strong growth inhibition and cell death efficacy for PC3 prostate cancer cells, and mimic 9, in which tyrosol (TYR) and homovanillyl alcohol (HVA) units were linked by a phosphodiester bond, was quite effective not only in cell growth inhibition but also in causing strong cell death under the study conditions and treatments that were not effective in PANC1 cells (194). Table 4 shows the biological activities of bioavailable curcumin and their mechanism.

**TABLE 4:** Therapeutics indications of curcumin, its impacts and mode of action.

Activity	Mode of action	References
Antioxidant	Enhance antioxidant capacity through increasing plasma superoxide dismutase (SOD) and catalase, as well as serum levels of glutathione peroxidase (GSH) and lipid peroxides.	(195)
	Elevate serum SOD.	(196)
	Act as scavenge various forms of free radicals, as reactive oxygen (ROS) and nitrogen species (RNS)	(197)
	Modulate GSH, catalase, and SOD in neutralization of free radicals.	(198)
	Inhibit ROS-generating enzymes like xanthine hydrogenase/oxidase and lipoxygenase/cyclooxygenase.	(199)
	Consider as lipophilic material, act as scavenger of peroxyl radicals, and act as a chain-breaking antioxidant.	(200)
Metabolic disorders	Improve insulin sensitivity.	(201)
	Suppress adipogenesis.	(202)
	Reduce elevated blood pressure.	(203)
	Modulate the expression of genes and enzymes activity included in lipoprotein metabolism causing lowering triglycerides and cholesterol levels in plasma.	(204)
	Elevate HDL-C concentrations.	(205)
	Decreases serum pro-inflammatory cytokines levels.	(206)
	Improve oxidative and inflammatory status.	(195)
Anti-inflammatory	Curcumin could block NF-κB activation elevated by various inflammatory stimuli.	(196)
	Curcumin has suppressing impact on NF-κB activation and induce the release of IL-8 and cell scattering that cause lowering in inflammation of stomach cells as the primary outcome for *H. pylori* in the gastric tissues. It suppresses the IκBα degradation, the activity of NF-κB DNA-binding and IκB kinase α and β (IKK α&β).	(207)
	Curcumin suppresses the matrix metalloproteinase-3 and metalloproteinase-9 activity (MMP-3 and MMP-9) as inflammatory molecules included in *H. pylori* in mice.	(208)
	Curcumin supplementation to the rats with *H. pylori*-induced stomach tissue inflammation caused a significant lowering in macromolecular leakage and NF-κB activation.	(209)
Arthritis	Improve osteoarthritis and rheumatoid arthritis via decreasing inflammation.	(210)
	Alleviate the symptoms of arthritis.	(211)
Cancer	Esophageal Cancer: curcumin modulated Notch-1 signaling and suppressed NF-jB and its downstream targets involving Bcl2, cyclin D1, VEGF, and MMP-9 in oral squamous cell carcinomas.	(212)
	Esophageal Cancer: curcumin reversed the bile acid induction of COX-2 and suppression of gene expression accompanied with sodium dis-mutase-1 in the esophageal HET-1A epithelial cell lines.	(213)
	Breast Cancer: curcumin down regulate of NF-jB, AP-1, COX-1, and –2, VEGF, fibroblast growth factor (FGF), cyclin E, IL-6, and –11, TGF-b, MMP-2, MMP-9, and MMP-13, the upregulation of tissue inhibitor of metalloproteinase-1.	(214)
	Breast cancer: curcumin reduced expression of Skp2, Her2, cyclin E, and CDK kinases in MDA-MB-231/Her2 cells.	(215)
	Brain cancers: curcumin stimulated both receptor-mediated and mitochondria-mediated proteolytic pathways for apoptosis in human glioblastoma T98G cells.	(216)
	Bone cancer: curcumin stopped cytokines and NF-jB but did not change p38 MAPK activation in the gingival tissues of experimental periodontal disease, *in vivo.*	(217)
	Thymic cancer: curcumin mediated thymic protection happens, at least in part, via neutralization of tumor-induced oxidative stress, restoration of NF-jB activity, and re-education of the TNF-signaling pathway.	(218)
	Pulmonary cancer: curcumin improved the expression of cancer suppressor DnaJ-like heat shock protein 40 (HLJ1) via JNK/JunD pathway activation and prevent human lung adenocarcinoma cell invasion and metastasis via modulating *E*-cadherin expression.	(219)
	Multiple Myeloma: curcumin prevents IL-6-induced STAT3 phosphorylation and subsequent STAT3 nu-clear translocation.	(118)
	Lymphoma: curcumin lowered NF-jB activity via ROS generation, subsequent by cyto-chrome c release and modulated Bax protein. It also activated caspase-9 and –3 and activated PARP cleavage revealing as-pase-dependent apoptosis in a Burkitt’s lymphoma cell line.	(220)
	Ovarian Cancer: curcumin prevents NF-jB and STAT3 activation and inactivate propagation of human ovarian cancer cell lines SKOV3ip1, HeyA8, and HeyA8-MDR, prevent ovarian cancers in athymic mice. In the SKOV3ip1 and HeyA8 *in vivo* models, curcumin lowered micro-vessel density and angiogenesis, and elevated cancer cell apoptosis.	(221)
	Leukemia: curcumin disrupted the G0/G1 phase of the tumor cell cycle and altered cell development via the upregulation of P27kipl, P21wafl, and pRb expression, and it downregulated cyclin D3. Curcumin stimulates apoptosis in CLL-B cells via the inactivation of STAT3, AKT, NF-jB, myeloid cell leukemia 1, and X-linked inhibitor of apoptosis protein, and it upregulated the proapoptotic protein BIM.	(222)
	Cervical Cancer: curcumin inhibits in a dose and time dependent the expression of E6 and E7 in cervical cancer cells. Curcumin prevented NF-jB activation via the suppression of IkBa phosphorylation and degradation, and down regulated the expression of COX-2 and AP-1 in cervical tumor cells.	(223)
	Bladder Cancer: curcumin induces apoptosis and inhibits human bladder cancer cells in the G2/Mphase by the downregulation of Bcl2, improvement of Bax and p53 expression. Curcumin stops the growth of urothelial cancers in a rat bladder carcinogenesis model.	(224)
	Renal Cancer; stimulate apoptosis in Caki cells via DNA fragmentation, the activation of cas-pase-3, cleavage of phospholipase C-g1, and the stimulation of ROS generation by the production of cytochrome c and downregulation of the Bcl2, Bcl-xL, IAP, and Akt pathways.	(225)
	Prostate Cancer: curcumin lower micro-vessel density in LNCaP prostate tumor cells, stop their proliferation and angiogenesis, *in vivo.*	(226)
	Pancreatic Cancer: curcumin lowers the expression of NF-jB-regulated gene products like COX-2, PGE2, and IL-8.	(227)
	Hepatic Cancer: curcumin lower the expression of Chk1 protein, stopped the cell cycle at the G2/M phase and stimulate apoptosis in hepatoma cell lines, stimulates DNA damage and growth arrest via elevation of ROS and lipid peroxidation in the therapy of HepG2 cells.	(228)
	Gastric Cancer: curcumin lower the expression of human epidermal growth factor receptor 2 and the activity of p21-activated kinase 1, stop the transformation of stomach cancer cells from the G1 to S phase via the downregulation of cyclin D1.	(229)
	Colorectal Cancer; curcumin therapy reveals cell shrinkage, chromatin condensation and DNA fragmentation by increasing growth arrest via pro-motion of the DNA damage-inducible gene (DDIT3) at the protein and mRNA levels in colon cancer cell lines, prevents the propagation of colon tumor cell lines and stimulates apoptosis by caspase-3-mediated b-catenin cleavage, downregulate c-Myc, blocks cell cycle progression by cyclin D and cyclin B expression, and elevated the activity of cell division control 2.	(230)
	Intestinal Cancer: curcumin lowered the expression of *b*-catenin in the red blood cell of Min/þ mice.	(231)
Antibacterial	*Curcuma longa* rhizome has antibacterial effect via the minimum inhibitory concentration (MIC) value of 4–16 g/L and minimum bactericidal concentration (MBC) value of 16 to 32 g/L versus *S. aureus*, *S. epidermis*, *Klebsiella pneumoniae* and *E. coli.*	(232)
	The hexane, ethanol turmeric extract and curcuminoids with 86.5% curcumin value, have antibacterial effect versus 24 pathogenic bacteria recovered from the chicken and shrimp.	(233)
	The adding of 0.3% (w/v) of aqueous curcumin extract to the cheese revealed lowering in bacterial numbers of *S. typhimurium*, *P. aeruginosa*, and *E. coli*. Moreover, it lowered the *S. aureus*, *B. cereus*, and *Listeria monocytogenes* pollution post 2 weeks of cold storage time.	(234)
	Curcumin has inhibitory activity on methicillin-resistant *S. aureus* strains with MIC value of 125–250 μg/mL.	(235)
	Curcumin has antibacterial effect with MIC values among 5 and 50 μg/mL versus 65 clinical isolates of *H. pylori.*	(236)
Antifungal	The adding the turmeric powder in plant tissue culture revealed that turmeric at the 0.8 and 1.0 g/L has inhibitory effect versus mycotic contaminations.	(237)
	The methanol extract of turmeric showed antimycotic effect versus *Cryptococcus neoformans* and *Candida albicans* with MIC values of 128 and 256 μg/mL, respectively.	(238)
	Curcumin has antifungal effect versus. *Aspergillus* and *Candida* species.	(239)
Antiviral	Di-*O*-tryptophanyl phenylalanine curcumin and di-*O*-decanoyl curcumin showed antiviral effect versus VSV and FIPV/FHV. However, bioconjugates did not show significant antiviral effect versus IIIB and ROD strains of type 1 human immunodeficiency virus (HIV-1) in MT-4 cells.	(240)
	Curcumin inactivates the acetylation of Tat protein of HIV accompanied with inhibition of (HIV-1) proliferation.	(241)
	Curcumin has antiviral activity via inhibition of coxsackievirus replication via dysregulation of the ubiquitin-proteasome system.	(242)

### Curcumin and exercise

Nutritional strategies, such as antioxidant-rich foods, which help regulate inflammation, immune function, and oxidative stress, may be beneficial during High-Intensity Interval Training (HIIT) (243). This HIIT exercise can increase the inflammatory response and hinder the body’s recovery process (140, 143, 244–247). Other supplements such as curcumin have emerged as agents with significant therapeutic potential. Curcumin supplementation improved recovery time in mice following an eccentric downhill running protocol in a model of exercise-induced muscle injury. Downhill running lowers both treadmill’s run time to fatigue and voluntary activity, while 10 mg of curcumin powder given three consecutive days before running offset these effects on performance. Lower creatine kinase, IL-6, and TNF-alpha concentrations were also observed in the soleus muscle (248). In a similar study, when a turmeric extract (100 mg/kg) was administered for 6 weeks to rats, the endurance time to exhaustion in the exercised group improved, and the concentrations of serum total cholesterol, high-density lipoprotein, triglycerides, and lactate levels in both the exercise and non-exercise groups improved (97). Similarly, in human models, other investigators have noticed that curcumin has the potential to prevent delayed-onset muscle soreness (DOMS) after downhill running and eccentric leg press exercises (249, 250).

In a human study, 20 healthy males took either 200 mg curcumin or a placebo twice a day for two days prior and 1-day after a 45-min, constant intensity (lactate 3.5-5 mmol/L) downhill run (grade –10%). The curcumin supplementation group experienced reductions in DOMS-related leg muscle pain at sites located on the anterior right thigh, posterior right thigh, anterior right leg, posterior right leg, anterior left thigh, posterior left thigh, anterior left leg, and posterior left leg. Magnetic resonance imaging (MRI) also revealed less muscle injury in both thighs’ posterior or medial compartments. The inflammatory marker, interleukin-8 (IL-8), was also significantly lower 2 h after the downhill running test contrasted to the placebo group (249).

In a similar experiment, 28 males and females were provided either a placebo or 400 mg/day of curcumin before completing eccentric-only dual-leg press exercises. They then ingested either curcumin or a placebo 2 days prior to and 4 days after the protocol. Curcumin was found to reduce creatine kinase on days 1–4 following eccentric exercise. Additionally, IL-8 was significantly decreased in the curcumin group on days 1 and 2 following the exercise, and TNF-alpha was significantly lower in the curcumin group on days 1, 2, and 4 following the exercise placebo (250). Similar effects have been observed with lower doses of curcumin, but some speculate that this may decrease the magnitude of the anti-inflammatory response. When participants ingested 150 mg curcumin prior and 12 h after eccentric contractions of the elbow flexors of one arm on an isokinetic dynameter, maximal voluntary contraction torque was preserved, and recovery occurred four days sooner post-exercise in the curcumin group when contrasted to a placebo-controlled group. Interestingly, no significant differences were found in creatine kinase, IL-6, or TNF-alpha, suggesting that a larger dose of curcumin and a greater frequency of consumption on recovery days (post-exercise) may be needed to have more significant effects (251).

In a similar study, participants ingested 200 mg of curcumin and 20 mg of piperine three times a day and then completed 25 repetitions of 25-m, one-leg jumps on a downhill slope. Concentric and isometric peak torque for knee extension, one-leg 6-second sprint performance, countermovement jump performance, muscle soreness, and creatine kinase concentrations were measured. The results showed a moderately lower sprint mean power output 24 h post-exercise in the curcumin group contrasted to the placebo group. The dose in this study was higher than what was used in the previous studies; the length of supplementation may have been too short to find differences that matter (252).

Few studies examined the potential of curcumin to enhance endurance performance. Eleven recreational athletes took 500 mg/day of curcumin or placebo in a randomized, cross-over design for three days before and on the day of the exercise trial. The trial consisted of 2 h on a cycle ergometer at a power output of 95% of their lactate threshold. Curcumin was associated with a reduced exercise-induced inflammatory response. More specifically, curcumin was linked with lower IL-6 concentrations one hour after exercise compared to placebo (253). The outcomes of these studies were that curcumin could affect performance and the inflammatory response throughout short-term interventions, but more well-controlled investigations are still needed.

### Curcumin and mental health

Curcumin is associated with better cognitive function and memory, as well as less stress and anxiety. Curcumin appears to be comparable with current pharmaceuticals as adjunctive therapy (254). Accumulating scientific evidence encourages using curcumin as a therapeutic agent for improving psychological health. In human studies, the most common psychological health assessments involve using questionnaires to quantify the signs and symptoms associated with mental health. In a sportive nutritional experiment, participants were supplemented with curcumin (3 days before and on the trial day) or placebo before completing a subjective daily analysis of life demands questionnaire to evaluate stress sources and stress signs before the exercise sessions. The curcumin group experienced “better than usual” results on the training days compared to the placebo on the second day of supply (253).

The obese patients witnessed by enhanced anxiety and depression, received 1,000 mg/day of a curcumin complex (curcumin, desmethoxycurcumin, and bisdemethoxycurcumin) or a placebo for 30 days. Curcumin treatment significantly reduced mean BAI scores compared to placebo, suggesting curcumin as an anti-anxiety therapy for individuals with obesity and major depressive disorder (255). Patients at a psychiatric outpatient department were enrolled in this study for 6 weeks. They were assigned to one of the three groups: 20 mg/day fluoxetine (*n* = 17), 1,000 mg/day curcumin (*n* = 10), or 1,000 mg/day and 20 mg/day of fluoxetine (*n* = 18). After 6 weeks of treatment, curcumin was equivalent to fluoxetine in terms of change in the Hamilton Depression Rating Scale score. The combination group showed a better response than the fluoxetine and curcumin groups alone. These results nominate curcumin as a powerful agent in treating major depressive disorder (256).

Cognition and memory are commonly explored in the older adult population. A computerized cognition test was used with 22 healthy older males and 38 healthy older females to evaluate the potential behavioral effects of curcumin. Participants received either curcumin (400 mg Longvida) or placebo once daily for four weeks and completed an array of computerized cognitive tasks preceding and following the assessment of the state of mood. A single dose of curcumin acutely improved cognitive processes and performance on a measure of sustained attention and working memory. After 4 weeks of curcumin supplementation, sustained attention and fatigue measures improved, proposing that curcumin may positively affect cognition in healthy elderly populations (254).

## The official indications of the joint food and agriculture organization of the united nations/world health organization expert committee on food additives

The Joint FAO/WHO Expert Committee on Food Additives (JECFA) is an international scientific expert committee that is administered jointly by the Food and Agriculture Organization of the United Nations (FAO) and the World Health Organization (WHO). A turmeric rhizome extract is authorized as food additive (color) under the name curcumin (E 100) in the EU [Commission Regulation (EU) No 1129/20116]. According to Commission Regulation (EU) No 231/20127, the following definition is allocated to this food additive: ‘Curcumin is obtained by solvent extraction of turmeric, i.e., the ground rhizomes of strains of *C. longa* L. In order to obtain a concentrated curcumin powder, the extract is purified by crystallization. The product consists essentially of curcumin; i.e., the coloring principle [1,7-bis(4-hydroxy-3-methoxyphenyl)hepta-1,6-dien-3,5-dione8] and its two desmethoxy derivatives 9 in varying proportions. Minor amounts of oils and resins naturally occurring in turmeric may be present. Only the following solvents may be used in the extraction: ethyl acetate, acetone, carbon dioxide, dichloromethane, *n*-butanol, methanol, ethanol, hexane, propan-2-ol.’ The curcuminoids, including methoxy curcumin, curcumin, and bisdemethoxycurcumin, are a group of compounds found in turmeric (17), authorized by the US Food and Drug Administration (FDA) as GRAS, “Generally Recognized As Safe” at doses between 4,000 and 8,000 mg/day (257).

JECFA assessed the food additive curcumin (turmeric rhizome extract) in 2003 and established an acceptable daily intake (ADI) of 0–3 mg/kg body weight (bw) (258). In the European Food Safety Authority (EFSA) Panel on Food Additives and Nutrient Sources added to Food (ANS) adopted a scientific opinion on the re-evaluation of the food additive color curcumin (E 100) (turmeric rhizome extract) and concluded that the available data set supports the ADI allocated by JECFA based on the NOAEL of 250–320 mg/kg bw per day from the reproductive toxicity study in rats for a decreased body weight gain in the F2 generation observed at the highest dose level, and an uncertainty factor of 100 (259, 260). The EFSA took into account additional information on the use of curcumin (E 100) in foods and carried out a refined exposure assessment (261).

The European Medicines Agency (EMA) (262) assessed *C. longa* L., rhizoma, as herbal medicinal product in the form of powdered herbal substance, comminuted herbal substance, dry extract [13–25:1, extraction solvent: ethanol 96% (v/v)], dry extract [5.5–6.5:1, extraction solvent: ethanol 50% (v/v)] and tinctures (1:5 or 1:10, extraction solvent: ethanol 70% (v/v).

## Applications of curcumin in food processing

Food preservation has been necessary for the continuity of life since the existence of humanity. Due to the changing eating habits over time and the increased number of employees, the development of ready-to-eat foods has become mandatory. Expanding food products’ shelf life and preserving their quality is a mandatory objective (263). The long-term preservation of seafood without spoiling is challenged by various requirements based on hygiene and sanitation rules, i.e., processing method, consuming seasons, processing wastes, making it ready to use the product and providing convenience for consumers and diversity for products. It has become important to utilize seafood processing in recent years.

Today, the increase in consumption and processing of food based on the relationship between the development of the industry and the consumption and production of food has made using food additives a technological must. The large number of people who work outside the home and do not have time to prepare food has encouraged the production of semi-finished or fully prepared foods commercially, and this situation has necessitated the use of food additives inevitable (264). Assuring food safety and security is one of the most important issues of today. Providing food security, improving food production, preventing nutrient losses, preserving their quality during the period between when the food is abundant and less, and extending its shelf life have gained significance. Thus, the usage of food additives has become inevitable in this case (5, 106, 265–271).

### One of the food additives: Colorants

The use of plants by humanity as paint dates back centuries. Therefore, dye plants have become the main dyestuff of industrial products such as textiles, food, and leather. There are nearly 150 plant species used in natural dye production. These plants are turmeric, elecampane, licorice, common juniper, and sage (4). Food dyes, a type of food additive, are used in the industry for various purposes, including preserving, elevating, or modifying the existing or typical color, controlling color change and deterioration, standardizing the appearance, adding decorative features, and creating new products. They are additives used in confectionery, food eaten between meals, soft drinks, pastries, and many foods such as gelatin desserts (272). Color is one of the first characteristics of food that attracts people. A conventional color is desired in foods to be consumed. Colorants are substances added in food production to correct changes in a color loss that occur during processing or at the end of the process, that is, to restore the color of food or to color food. Color substances are also essential for creating a standard pigment in the product technologically (273).

The colorants are used to regain the natural color lost during processing and storage, to strengthen the weak color, to color the colorless food, and to meet consumer acceptance by hiding low quality (274). Since the additives are chemicals, their excess is harmful to health. Excessive use of these colorants may endanger one’s health. According to the way they are obtained, the colorants are divided into two natural and artificial colorants. Natural colorants are obtained from microbial, vegetable, animal, and mineral sources. The color stability of natural colorants is very low against physical and chemical effects. The majority of natural colorants are low in water solubility (275). According to the way they are obtained, the colorants are divided into two natural and artificial colorants. Natural colorants are obtained from microbial, vegetable, animal, mineral sources and agricultural wastes which are rich sources of natural colorants such as anthocyanin canthaxanthin, plain caramel, carotenes and chlorophylls (4, 276). Artificial colorants are more preferred in the food industry in terms of their physicochemical properties. Artificial colorants are easily soluble in water and oil (277). Curcumin is a powerful coloring agent widely used in the food industry. Its extraction from the plant *C. longa* is commonly done with aqueous solvent solutions. In contrast to the conventional extraction methods, two different green and bio-based surfactant-free microemulsion (SFME) extraction systems, which were approved for food and yield a higher extracting power of curcuminoids were compared. Two SFMEs, water/ethanol/triacetin and water/diacetin/triacetin, were investigated via dynamic light scattering. Curcumin solubility in binary mixtures consisting of ethanol/triacetin or diacetin/triacetin was studied both experimentally and theoretically using UV–Vis measurements and COSMO-RS. The SFMEs were further examined and compared to a common ethanol/water (80/20) extraction mixture with respect to their extracting ability using high performance liquid chromatography. The SFMEs containing ethanol were found to extract ∼18% more curcuminoids than the SFMEs containing diacetin and ∼53% more than the ordinary ethanol/water mixture (278). Also, (279) observed that using turmeric instead of Sunset yellow FCF can provide positive changes in the product’s appearance, taste, and texture that may appeal to the consumer. It has also been found to impact the increased consumption of smoked garfish positively. Thus, the use of turmeric, which is a natural colorant where more positive results are obtained, instead of artificial colorants that may be harmful to human health, has been suggested in terms of both making the color that is impulsive to the consumer in the product attractive and increasing the consumption of the smoked product (279).

### Turmeric, one of the spices used in foods

Spices are usually cooked with food, but microorganisms in the form of spores remain alive during the cooking process, causing a proliferation in products stored in improper conditions during storage and distribution. The microflora in the spice reduces product shelf life and causes spoilage and foodborne diseases. These bacteria generally cause spoilage in products such as pickles, salami, sausage, and canned food. Turmeric is obtained from the *C. longa* plant’s root, a ginger family fiber plant. It is a plant with polyphenolic characteristics (279).

Dried turmeric is applied as a spice, and in making curry, it gives the curry a yellow color. With its bright yellow color, turmeric has been utilized as paint, medicine, and a spice since the 600s BC. Marko Polo has described turmeric as a vegetable that replaces saffron but is not saffron (279).

Turmeric is added in smoked foods, pickles, and some cakes. It is used in some dishes, the mixture of curry, mustard, and sauces for chicken meat, in some desserts, especially zerde, a dessert served at weddings in Anatolia, and gives it its yellow color. It is also used in seafood, fish soup, egg dishes, soups, rice, cold cuts, and numerous vegetable dishes. It is used especially in Indian and South Asian cuisine (279).

The scarce *in vivo* study explored the healthy benefits of curcumin in a functional food (280), which evaluated the effect of curcumin in combination with phytosterols in bread on the lipid profile. No significant effect was observed for curcumin addition in bread, whereas the incorporation of phytosterols reduced plasma cholesterol levels. Additionally, the authors indicated that this lack of effect observed for curcumin could be explained by the high temperatures used to process bread, which favored curcumin degradation. *In vitro* study of antimicrobial and antioxidant gelatin/curcumin composite films for active food packaging application, the authors found the addition of 1.5% of curcumin improved the UV blocking effect by more than 99% at a loss of 5.7% of transparency compared to neat gelatin films. The addition of curcumin (up to 1 wt%) significantly improved mechanical and water vapor barrier properties. Also, the gelatin/curcumin composite films exhibited remarkable antimicrobial activity against foodborne pathogenic bacteria, *E. coli* and *L. monocytogenes*, and showed strong antioxidant activity comparable to ascorbic acid. Antibacterial and antioxidant gelatin/curcumin composite films with improved UV protection, water vapor barrier and mechanical properties have high potential in active food packaging applications (281). Also, another *in vitro* study (282), found that poly lactic acid (PLA)/curcumin composite film showed excellent antioxidant and some antibacterial activity. The functional PLA/curcumin composite films with improved physical and functional properties can be used in active food packaging applications (282).

It is also worth mentioning that the incorporation of turmeric extract in beverages, bread, biscuits, snacks, pasta, milk, cheese, fresh sausage, and patties has been studied. Al-Obaidi (283) evaluated the influence of turmeric powder on the chemical composition, oxidative stability, and microbiology of the soft cheese. Different concentrations of turmeric powder (0, 0.1, 0.2, and 0.3%) represented as (T1, T2, T3, and T4) were added to the milk processed to the soft cheese, and then the cheese produced was stored at 5 ± 2°C for 9 days. The results showed no significant differences between the cheese of the different treatments (T2, T3, and T4) and the control cheese (T1) for moisture, protein, fat, ash, and pH. Evaluations of the cheese’s color, texture, and bitterness showed no significant differences between the cheese treated with turmeric powder (T2, T3, and T4) and the control cheese (T1). While for the flavor, the results showed that there was a significant difference between the cheese samples treated with turmeric powder 0.3% (w/v) (T4) and the untreated cheese sample (control) (T4). The results of peroxide value (PV) and acid value (AV) showed that the cheese treated with different concentrations of turmeric powder was lower than untreated cheese (control) (283). The microbiological results indicated that as the concentration of the turmeric powder increased, the total bacterial count decreased compared with the control treatment, which showed the highest total count after 9 days of storage at 5 ± 2°C. However, the coliform bacteria were increased during storage for control treatment only while it was undetectable for T2, T3, and T4 treatments after storage at 5 ± 2°C for 9 days. It could be concluded that turmeric powder was successfully used to improve the keeping quality of soft cheese (283).

Also, de Carvalho et al. (284) evaluated the *in vitro* effect of turmeric extract as a natural antioxidant on modified atmosphere-packaged fresh lamb sausages with fat replacement during storage (2°C). Five treatments were prepared: control without antioxidant (CONT); with 500 mg/kg sodium erythorbate (E500); and three batches with 250, 500, or 750 mg/kg turmeric extract (T250, T500, and T750). Turmeric extract improved the antioxidant capacity of lamb sausages and also slowed lipid oxidation and the generation of related volatile compounds. Moreover, the physicochemical parameters of lamb sausages were not greatly influenced by turmeric addition and concentration, except for the yellow color. All samples were considered acceptable by consumers. These findings showed that turmeric extract is effective against lipid oxidation and could be a good strategy to enhance the shelf life of lamb sausage (284).

Furthermore, Gómez-Estaca et al. (285) studied the effect of household practices (chilled storage and cooking) on quality attributes of low-fat PUFA-enriched pork burgers and the presence of curcumin as an antioxidant. Curcumin effectively reduced the lipid oxidation process derived from chilled storage or cooking regardless of the oleogelation method; the resulting products showed a lipid composition that meets recommendations for fatty acid intake. The samples formulated with beeswax oleogel presented adequate technological properties and overall sensory acceptability. Curcumin provided a yellow color that reduced sensory acceptance, regardless of the oleogelation method. Further studies are needed to adapt ethyl cellulose oleogels prepared with highly unsaturated lipids for the successful development of fresh meat products due to the extent of lipid oxidation during refrigeration and cooking and to the lower sensory acceptability.

Additionally, Ribeiro Oliveira et al. (286) developed extruded snacks from broken rice grains (BRG) and turmeric powder (TP). Snacks were *in vitro* evaluated for their physicochemical, microbial, and sensory characteristics and the selected savory snack formulation’s chemical composition, phenolic compounds, and antioxidant capacity. The selected snack, with the physical characteristics closest to the traditional corn product, was obtained with 6% substitution of BRG by TP and was constituted by 7.76% protein, 4.78% lipid, 5.84% dietary fiber, 75.3% available carbohydrates, and 174.75 mg of gallic acid equivalent per 100 g and 6.52% of DPPH scavenging capacity. The selected snack was shown to be feasible once TP was added as an ingredient that aggregated sensory, nutritional, and functional (antioxidant) values to the BRG-based snack, providing an alternative for the production of gluten-free snacks (286).

Three freshwater fish products (shell, taki, and tengra) were examined for their physicochemical (physical properties, proximate and chemical analyses), mineral, and bacteriological quality using turmeric powder, salt (dry), and the sun-drying procedure. The physicochemical properties such as the sensory properties, moisture, protein, fat, ash, salt, TVB-N, FFA, pH, certain mineral contents (Ca, Mg, Fe, Cu, Zn, and Mn), and bacterial load (SPC and HBC) of freshly processed turmeric and salt-treated sun-dried fish were examined. The dried fish product with the least moisture was also the most resistant to enzyme and microbiological activity (287).

Raw meat samples from pigs fed a control diet and a diet mixed with 4.5 g of turmeric powder per pig daily were used. Following the slaughter, raw meat was kept at 4°C for 7 days, and it was concluded that dietary turmeric powder induced an elevation in cooked meat quality, and color modifications in cooked meat were correlated with color parameters of raw samples. Dietary supplementation with *C. longa* powder did not change lipid oxidation, Warner Bratzler shear force, or the antioxidant capacity of cooked meat (288). In another study, the impacts of turmeric powder and ascorbic acid on lipid oxidation and antioxidant capacity in cooked rabbit burgers were estimated. The burgers were derived from 3 different formulations (C, control, with no additives; Tu with 3.5% of turmeric powder and AA with 0.1% of ascorbic acid) and were stored at 4°C for 0–7 days and cooked, and they concluded that the addition of 3.5% of turmeric powder in rabbit burgers exerts an antioxidant impact during storage. It appears more potent in limiting lipid oxidation than ascorbic acid post-cooking (289).

## Curcumin usage limitation and future plans

Curcumin poses potential health risks despite its numerous advantages for human health and its well-established safety profile. Individuals’ side effects observed include abdominal pain, nausea, constipation, and hot flashes (290, 291). According to some accounts, curcumin may cause toxicity under certain circumstances. For instance, individuals receiving doses of curcumin between 0.45 and 12 g may suffer from vomiting, diarrhea, headaches, rushes, yellow stools, and elevated levels of both blood alkaline phosphatase and lactate dehydrogenase (292). For example, curcumin appears to cause DNA damage at both the mitochondrial and nuclear levels, suggesting that it may have a carcinogenic effect at doses near those that show positive results.

Additionally, mice fed different doses of turmeric oleoresin for 3 months and 2 years showed carcinogenic potential (292). Unsaturated ketones appear to be related to the mechanisms causing these effects, and these regions are known to form covalent bonds via a Michael addition reaction with the thiol groups of cysteine residues, and this also could be due to the antioxidant enzyme thioredoxin reductase that may be altered by this method, resulting in the formation of ROS (293). It can also inhibit the “guardian of the genome” “p53” and cause DNA damage caused by topoisomerase II. Curcumin was also discovered to inhibit the activities of cytochrome P450, glutathione S-transferase, UDP-glucuronosyltransferase, and chelate iron, which has an impact on how it is metabolized throughout the body. As xenobiotics build up when these three drug-metabolizing enzymes are blocked, toxicity may result (293). Numerous experiments on healthy subjects have confirmed the safety and effectiveness of curcumin. Despite this well-established safety, some negative side effects have been reported. Seven subjects receiving 500–12,000 mg in a dose-response study and followed for 72 h experienced diarrhea, headache, rash, and yellow stool (93). In another study, some subjects receiving 0.45–3.6 g/day curcumin for 1–4 months recorded nausea and diarrhea and elevated serum alkaline phosphatase and lactate dehydrogenase contents (294).

## Conclusion

Curcumin has a prolonged history of usage in ancient medicine, where it was used in various medical treatments as well as food coloring and spice. Science has advanced over time, demonstrating curcumin’s many positive benefits for human health. The “golden spice” is still used in cooking today. Still, technological advances have made it possible to employ curcumin for various uses in the food and health industries. According to the results of preclinical and clinical studies conducted *in vitro* and *in vivo*, respectively, curcumin may be helpful in the prevention and treatment of many diseases, including cardiovascular diseases, diabetes mellitus, obesity, allergy, asthma, inflammatory diseases, and neurodegenerative disorders, e.g., Alzheimer’s, Parkinson’s, multiple sclerosis, and Huntington’s disease by affecting different molecular targets. Compared to other medications, curcumin is viewed as a very cost-effective and safe natural substance that can be used to prevent and treat many disorders. According to results from clinical trials, nano-formulations can increase curcumin bioavailability and are systemically safe. Nevertheless, these nano-formulations must be tested as therapeutic modalities for impending clinical studies and human application. Additionally, using curcumin nano-formulations in combination with other drugs is a good way to lower the dose of the primary therapeutic ingredient, which can increase therapeutic effectiveness while lowering systemic toxicity. Numerous curcumin nano-formulations have been developed, including dendrimers, polymeric nanoparticles, nanocrystals, micelles, liposomes, SLNs, and nanogels. These formulations are being investigated in several experimental and clinical studies and are suspected of being responsible for a few neurodegenerative diseases. Further research is recommended to assess the side impacts of different curcumin nano-formulations on human health and environmental significance.

## Author contributions

All authors listed have made a substantial, direct, and intellectual contribution to the work, and approved it for publication.
